# Diagnostic utility and tissue localization of HPV16 and HPV18 E7 immunohistochemistry in cervical lesion progression: a comparative study with PCR

**DOI:** 10.3389/fonc.2026.1942646

**Published:** 2026-09-10

**Authors:** Fatimah Suliman Mohammad Aljebaly

**Affiliations:** Department of Pathology, College of Medicine, Qassim University, Buraydah, Qassim, Saudi Arabia

**Keywords:** cervical cancer, cervical intraepithelial neoplasia, cervical lesions, diagnostic pathology, E7 oncoprotein, HPV16, HPV18, immunohistochemistry

## Abstract

**Background:**

Persistent infection with high-risk human papillomavirus (HPV), particularly HPV16 and HPV18, contributes to cervical carcinogenesis through sustained expression of the viral E7 oncoprotein. Although HPV DNA polymerase chain reaction (PCR) is widely used for viral detection, it does not directly reflect biologically active oncogenic transformation within tissue. This study evaluated the diagnostic utility, clinicopathological significance, tissue localization, and concordance of HPV16 and HPV18 E7 immunohistochemistry (IHC) across the spectrum of cervical lesion progression.

**Methods:**

A retrospective study was conducted on 109 formalin-fixed paraffin-embedded cervical tissue specimens representing non-neoplastic, premalignant, and malignant lesions. HPV16 and HPV18 E7 protein expression was assessed by immunohistochemistry and compared with HPV DNA PCR findings. Concordance analysis, receiver operating characteristic (ROC) analysis, and multivariable logistic regression were performed to evaluate diagnostic performance and independent predictive value.

**Results:**

HPV16 and HPV18 E7 expression increased progressively with lesion severity, with the highest immunoreactivity observed in high-grade squamous intraepithelial lesions and invasive carcinoma. Substantial concordance was observed between IHC and PCR findings for both HPV types. HPV16 demonstrated superior diagnostic performance, with sensitivity, specificity, accuracy, and area under the curve (AUC) values of 92.3%, 83.9%, 89.9%, and 0.925, respectively, compared with HPV18 (90.0%, 79.3%, 83.7%, and 0.894). Multivariable logistic regression identified HPV16 and HPV18 E7 expression as significant independent predictors of disease status. Histopathological evaluation demonstrated progressive localization patterns, transitioning from focal basal/parabasal staining in low-grade lesions to diffuse full-thickness epithelial and tumor-associated expression in high-grade lesions and invasive carcinoma.

**Conclusions:**

HPV16 and HPV18 E7 immunohistochemistry demonstrated strong association with cervical lesion severity and substantial concordance with PCR findings, supporting its potential utility as a complementary tissue-based biomarker in cervical pathology. The findings further suggest that E7 immunohistochemistry may provide clinically relevant information regarding biologically active HPV-driven transformation and improve lesion characterization in diagnostically challenging cases.

## Graphical abstract

**Figure:**
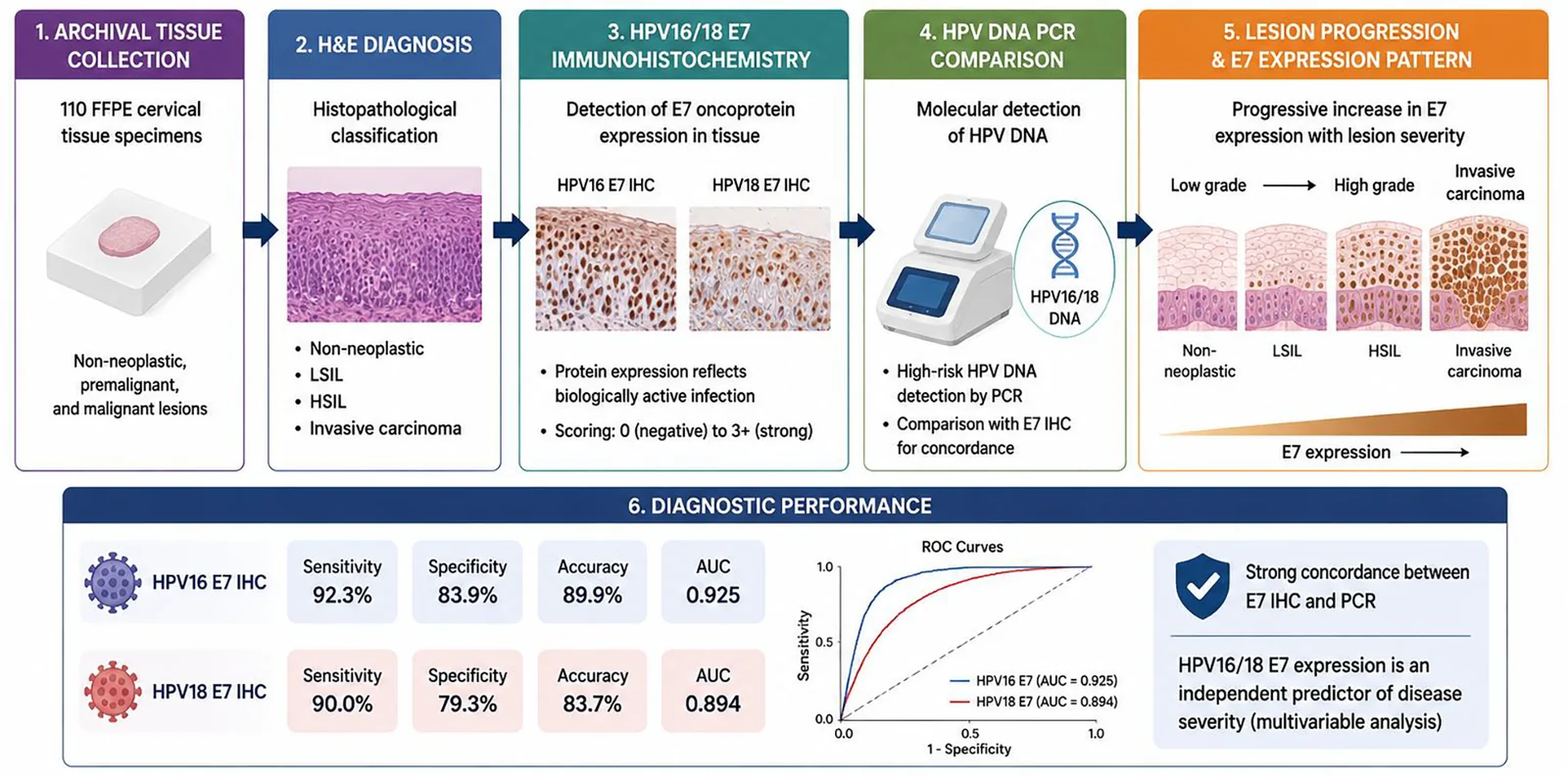

## Introduction

Cervical cancer remains one of the most significant causes of cancer-related morbidity and mortality among women worldwide, particularly in low- and middle-income countries where access to organized screening and molecular diagnostic infrastructure remains limited (1–3). Despite substantial advances in vaccination and screening strategies, cervical cancer continues to impose a considerable global health burden, largely due to delayed diagnosis and persistent infection with high-risk human papillomavirus (HPV) genotypes (4–7). Among these, HPV16 and HPV18 are among the most clinically important high-risk HPV genotypes implicated in cervical carcinogenesis, contributing substantially to cervical dysplasia, malignant transformation, and disease-related morbidity worldwide. Despite advances in molecular HPV detection, tissue-based biomarkers reflecting biologically active viral oncogenesis remain incompletely characterized (3, 8, 9).

The malignant transformation associated with high-risk HPV infection is primarily driven by the continuous expression of the viral oncoproteins E6 and E7 (10). These oncoproteins disrupt critical tumor suppressor pathways through functional inactivation of p53 and retinoblastoma (pRb) proteins, resulting in uncontrolled cellular proliferation, genomic instability, and progressive epithelial dysplasia (11, 12). In particular, the E7 oncoprotein plays a central role in promoting cell cycle dysregulation and sustaining oncogenic progression, making it a biologically relevant target for diagnostic evaluation (10). Unlike transient HPV infection, persistent expression of E7 is more closely associated with transforming infections and disease progression, suggesting potential utility beyond simple viral detection (13, 14).

Current cervical cancer screening and diagnostic approaches rely predominantly on cytology, histopathology, HPV DNA testing, and surrogate biomarkers such as p16INK4a (15, 16). Although HPV DNA-based molecular assays are highly sensitive for detecting viral presence, they do not necessarily distinguish transient infection from biologically active oncogenic transformation (3, 17, 18). Similarly, histopathological interpretation may occasionally be challenging in morphologically equivocal lesions, particularly in cases with overlapping reactive and dysplastic features (19). While p16 immunohistochemistry has improved diagnostic reproducibility in cervical pathology, it represents an indirect surrogate marker of HPV-mediated transformation rather than direct evidence of viral oncoprotein activity (20, 21).

Increasing attention has therefore been directed toward HPV E6/E7-targeted biomarkers because of their closer biological association with active carcinogenic transformation. Among these, E7 plays a pivotal role in cell cycle dysregulation through disruption of retinoblastoma protein (pRb)-mediated signaling and sustained epithelial proliferation (22). Immunohistochemical detection of HPV E7 offers the additional advantage of preserving tissue architecture while allowing direct visualization of viral oncoprotein localization within lesional epithelium, thereby providing potentially meaningful clinicopathological information beyond molecular detection alone (23).

Despite increasing interest in HPV-related biomarkers, the clinicopathological significance, diagnostic performance, and tissue localization patterns of HPV16 and HPV18 E7 immunohistochemistry across cervical lesion progression remain incompletely characterized, particularly in direct comparison with PCR-based molecular detection. Furthermore, limited evidence exists regarding the potential utility of E7 immunohistochemistry as a complementary tissue-based biomarker in diagnostically challenging cervical lesions.

Therefore, the present study aimed to evaluate the diagnostic utility, clinicopathological significance, and tissue localization of HPV16 and HPV18 E7 immunohistochemical expression across the spectrum of cervical lesions. In addition, concordance between immunohistochemistry and HPV DNA PCR was assessed, together with diagnostic performance and independent predictive value in relation to disease severity. By integrating tissue-based and molecular approaches, this study seeks to provide further evidence supporting the potential role of HPV E7 immunohistochemistry as a complementary biomarker in cervical pathology.

## Materials and methods

### Study population

This retrospective study included archived formalin-fixed paraffin-embedded (FFPE) cervical tissue specimens retrieved from the Department of Pathology, College of Medicine, Qassim University, Saudi Arabia. The study cohort comprised histologically confirmed cervical lesions representing non-neoplastic, premalignant, and malignant categories of cervical pathology. For comparative molecular evaluation, only cases with available HPV DNA polymerase chain reaction (PCR) results were included.

Eligible cases fulfilled the following inclusion criteria: confirmed histopathological diagnosis, availability of adequate FFPE tissue for immunohistochemical evaluation, complete clinicopathological information, and satisfactory tissue preservation. Cases with incomplete clinical records, inadequate tissue material, poor preservation, or technically unsatisfactory staining quality were excluded to ensure analytical reliability and diagnostic accuracy.

The specimens were obtained from women who underwent cervical biopsy or excision because of abnormal cervical cytology, clinical suspicion of cervical disease, or histopathological evaluation of a cervical lesion. Patients were managed through the relevant gynecological and/or specialist clinical services associated with the participating institution, and tissue specimens were subsequently referred to the Department of Pathology for routine histopathological diagnosis. The final study cohort included cases spanning benign cervical changes, low- and high-grade squamous lesions, adenocarcinoma *in situ*, and invasive squamous and glandular carcinomas.

### Study period and specimen retrieval

This retrospective study included archived formalin-fixed paraffin-embedded cervical tissue specimens collected between 2014 and 2024 at the Department of Pathology, College of Medicine, Qassim University, Saudi Arabia. Histopathological diagnosis and HPV molecular testing were performed as part of the original clinical evaluation, while the immunohistochemical analysis was performed retrospectively during June 2025 – December 2025.

### Sample size

A total of 109 archived cervical tissue specimens fulfilled the study eligibility criteria and were included in the final analysis. The cohort encompassed a spectrum of cervical lesion categories ranging from non-neoplastic lesions to invasive carcinoma (**Table 1**), enabling comparative evaluation of HPV16 and HPV18 E7 immunohistochemical expression across different stages of disease progression.

**Table 1:** Baseline clinicopathological characteristics of study participants (n = 109).

Variable	Category	n (%)
Histopathological diagnosis	AIS	2 (1.8)
	ASC-US/ASC-H	4 (3.7)
	Adenocarcinoma (AC)	10 (9.2)
	HSIL	34 (31.2)
	Koilocytotic change	1 (0.9)
	LSIL	26 (23.9)
	NILM	11 (10.1)
	SCC	19 (17.4)
	Squamous metaplasia	2 (1.8)
Age (years)	Mean ± SD	50.7 ± 10.1
	Median (IQR)	53 (41–59)

NILM, negative for intraepithelial lesion or malignancy; ASC-US, atypical squamous cells of undetermined significance; ASC-H, atypical squamous cells—cannot exclude HSIL; LSIL, low-grade squamous intraepithelial lesion; HSIL, high-grade squamous intraepithelial lesion; AIS, adenocarcinoma *in situ*; SCC, squamous cell carcinoma; AC, adenocarcinoma; SD, standard deviation; IQR, interquartile range.

The sample size permitted assessment of clinicopathological associations, concordance between immunohistochemistry and HPV DNA PCR, receiver operating characteristic (ROC) curve analysis, and multivariable logistic regression modeling for evaluation of diagnostic performance and independent predictive value.

### Immunohistochemical staining

Immunohistochemical (IHC) staining was performed on selected FFPE cervical tissue sections to evaluate the expression of HPV16 and HPV18 E7 oncoproteins. Tissue sections were cut at 4 µm thickness using a rotary microtome and mounted onto positively charged glass slides.

The tissue sections were deparaffinized in xylene and subsequently rehydrated through descending concentrations of ethanol at room temperature. Heat-induced antigen retrieval was carried at 100 °C using Tris-EDTA buffer (pH 9.0) for 40 minutes. The sections were incubated overnight at 4 °C with the following primary antibodies:

- Santa Cruz^®^ HPV16 E7 (sc-6981; dilution 1:50)
- Santa Cruz^®^ HPV18 E7 (sc-365035; dilution 1:50)

Following primary antibody incubation, the slides were treated with mouse LINKER (Dako, Denmark) and subsequently incubated with horseradish peroxidase (HRP)-conjugated secondary antibody (Dako, Denmark) at room temperature. Immunoreactivity was visualized using 3,3′-diaminobenzidine (DAB) substrate solution as the chromogen, followed by hematoxylin counterstaining.

The stained sections were dehydrated through ascending ethanol concentrations, cleared in xylene, and permanently mounted for microscopic evaluation.

### Positive and negative controls

Appropriate controls were included in each immunohistochemical staining batch to ensure staining specificity, reproducibility, and quality assurance. Since all selected cervical tissue specimens had previously undergone HPV16 and HPV18 DNA testing by PCR, thyroid tissue confirmed to be negative for HPV16 and HPV18 DNA were used as negative tissue controls and demonstrated absence of specific immunoreactivity. In addition, negative reagent controls were prepared by omitting the primary antibody during the staining procedure to further exclude nonspecific staining.

Cervical carcinoma tissues with previously confirmed HPV16 and HPV18 DNA positivity by PCR were used as positive controls and demonstrated strong immunoreactivity. However, it should be noted that PCR-based detection confirms the presence of viral DNA and does not necessarily indicate active viral oncoprotein expression at the protein level. Therefore, positive PCR status may not always directly correlate with the intensity of immunohistochemical protein expression observed within tissue sections.

### Interpretation and scoring of immunohistochemical expression

Immunohistochemical interpretation was independently performed by three researchers experienced in histopathological evaluation. Discordant findings were resolved by consensus, with adjudication by a fourth independent reviewer when necessary.

Cytoplasmic staining with or without nuclear positivity was considered indicative of HPV E7 expression. Staining intensity was graded as follows: 0 (absent), 1 (mild), 2 (moderate), and 3 (strong). The proportion of positively stained cells was scored as 0 (0%), 1 (<10%), 2 (10–50%), 3 (51–80%), and 4 (>80%).

An immunoreactive score (IRS) ranging from 0 to 12 was calculated by multiplying staining intensity and percentage positivity scores. Expression was categorized as negative (0–1), mild (2–3), moderate (4–8), or strong (9–12).

Particular attention was given to tissue localization patterns across lesion grades, including basal/parabasal epithelial staining, full-thickness epithelial expression, and staining within invasive tumor nests.

For the primary diagnostic analysis, IHC staining was dichotomized as positive when any specific staining intensity was observed (mild, moderate, or strong), while complete absence of staining was classified as negative. To assess the effect of the positivity threshold on diagnostic performance, a prespecified sensitivity analysis was additionally performed using a more stringent definition in which only moderate and strong staining were considered positive, whereas mild and negative staining were considered negative.

Inter-observer agreement was assessed among the three independent observers using Fleiss’ kappa, which is appropriate for categorical ratings by more than two observers. Agreement was evaluated across the four IHC staining categories (Negative, Mild, Moderate, and Strong). Cases with incomplete observer ratings were excluded from the corresponding agreement analysis. In addition to Fleiss’ kappa, the proportion of cases in which all three observers assigned the same staining category was calculated as the exact percentage agreement.

### HPV DNA extraction and PCR

HPV DNA testing was performed as part of the original clinical laboratory evaluation of the cervical specimens. Information regarding HPV DNA testing, including the testing laboratory, assay used, and HPV genotype results, was retrospectively retrieved from the laboratory register and corresponding clinical records. HPV testing was performed either at the study institution or at external diagnostic laboratories, depending on the original clinical referral and testing pathway. Therefore, the HPV PCR assays, DNA extraction methods, primer sets, amplification conditions, and assay platforms were not uniform across all specimens. The present study used the recorded HPV16 and HPV18 DNA PCR results as the available molecular reference results for comparison with HPV16 and HPV18 E7 immunohistochemistry. Where available, the laboratory records were reviewed to verify the assay and testing information associated with individual specimens.

Because this was a retrospective study based on archived specimens and previously performed clinical testing, complete technical information was not available for every PCR result. Where documented in the laboratory register, the assay platform, target genotype(s), and relevant testing information were recorded. For specimens tested at external laboratories, the original laboratory-specific technical protocol was not available to the investigators in all cases. We therefore did not infer or retrospectively assign primer sequences, amplicon sizes, or cycling conditions where these were not documented in the original laboratory records. This heterogeneity was considered when interpreting concordance between HPV DNA PCR and E7 immunohistochemistry.

### Concordance and diagnostic performance analysis

Concordance between immunohistochemistry and PCR findings for HPV16 and HPV18 was evaluated by categorizing cases as concordantly positive, concordantly negative, PCR-positive/IHC-negative, or PCR-negative/IHC-positive.

Diagnostic performance indices including sensitivity, specificity, accuracy, and area under the receiver operating characteristic curve (AUC) were calculated to assess the predictive performance of HPV16 and HPV18 immunohistochemical expression relative to PCR findings.

### Statistical analysis

Statistical analyses were performed using R statistical software (R Foundation for Statistical Computing, Vienna, Austria). Descriptive analyses were used to summarize demographic and clinicopathological characteristics.

Receiver operating characteristic (ROC) curve analysis was performed to evaluate the diagnostic performance of HPV16 and HPV18 E7 immunohistochemistry relative to PCR findings. Sensitivity, specificity, accuracy, and area under the curve (AUC) were calculated.

Multivariable logistic regression analysis: Associations between E7 IHC and the corresponding HPV PCR result were evaluated using multivariable logistic regression. Because the histopathological diagnosis score represented an ordinal measure of lesion severity and its treatment as a continuous linear predictor was not considered sufficiently justified, it was not retained in the final model. Age was included as a covariate. Potential multicollinearity was assessed using variance inflation factors (VIFs). Because the initial conventional logistic regression models showed evidence of sparse-data instability and potential separation, Firth’s penalized maximum-likelihood logistic regression was used for the final multivariable analyses. Odds ratios (ORs) with 95% confidence intervals (CIs) and two-sided P values were reported.

## Results

### Age distribution across diagnostic categories

**Figure 1:**
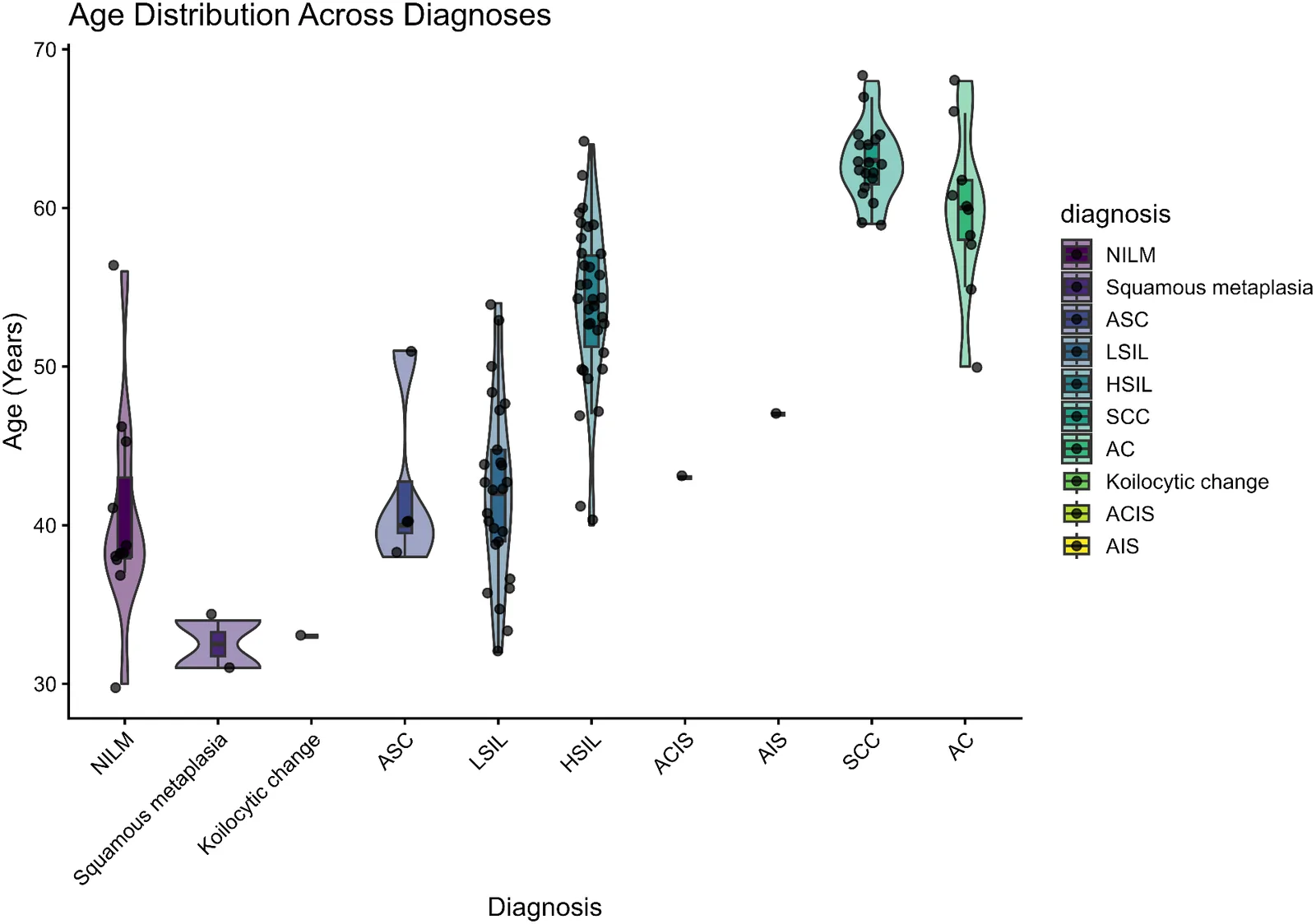
Age distribution across diagnostic categories is shown using a violin/box plot stratified by cytological and histopathological diagnosis. Categories include NILM (negative for intraepithelial lesion or malignancy), benign changes (squamous metaplasia and koilocytotic change), atypical squamous cells (ASC-US/ASC-H), low-grade squamous intraepithelial lesion (LSIL), high-grade squamous intraepithelial lesion (HSIL), squamous cell carcinoma (SCC), adenocarcinoma (AC), adenocarcinoma *in situ* (AIS).

The age distribution across diagnostic groups is illustrated in **Figure 1**. A clear increasing trend in age was observed across disease severity categories. Patients with NILM (negative for intraepithelial lesion or malignancy) and benign conditions such as squamous metaplasia and koilocytotic change were generally younger, with relatively narrow age distributions.

In contrast, intermediate lesions such as ASC-US/ASC-H (atypical squamous cells) and LSIL (low-grade squamous intraepithelial lesion) showed a broader age spread, with a moderate upward shift in median age compared with normal cytology groups. Patients diagnosed with HSIL (high-grade squamous intraepithelial lesion) demonstrated further age elevation, indicating progression toward higher-grade disease in older individuals.

The highest age distributions were observed in invasive cancer groups, particularly SCC (squamous cell carcinoma) and adenocarcinoma (AC), where patients clustered predominantly in older age ranges (approximately 55–70 years). AIS (adenocarcinoma *in situ*) also showed relatively elevated age profiles, consistent with premalignant-to-malignant transition patterns. The correlation matrix demonstrates similar results (**Supplementary Figure 1**).

### Inter-observer agreement

The three observers demonstrated high agreement in the assessment of HPV E7 IHC staining. For HPV16 E7, 109 cases had complete ratings from all three observers, with an exact agreement of 86.79% and a Fleiss’ kappa of 0.880 (p < 0.001). For HPV18 E7, all 109 cases had complete ratings, with an exact agreement of 92.66% and a Fleiss’ kappa of 0.912 (p < 0.001) (**Table 2**). These findings indicate high reproducibility of the IHC scoring across observers.

**Table 2:** Inter-observer agreement for HPV16 and HPV18 E7 IHC scoring.

IHC marker	Number of observers	Complete cases, n	Exact agreement, %	Fleiss' κ	p-value
HPV16 E7	3	109	86.79	0.880	<0.001
HPV18 E7	3	109	92.66	0.912	<0.001

κ, Fleiss' kappa. Agreement was calculated across the four staining categories: Negative, Mild, Moderate, and Strong. Exact agreement represents the percentage of cases for which all three observers assigned the same staining category.

### Association between HPV16/18 PCR status and cervical lesion severity

**Figure 2:**
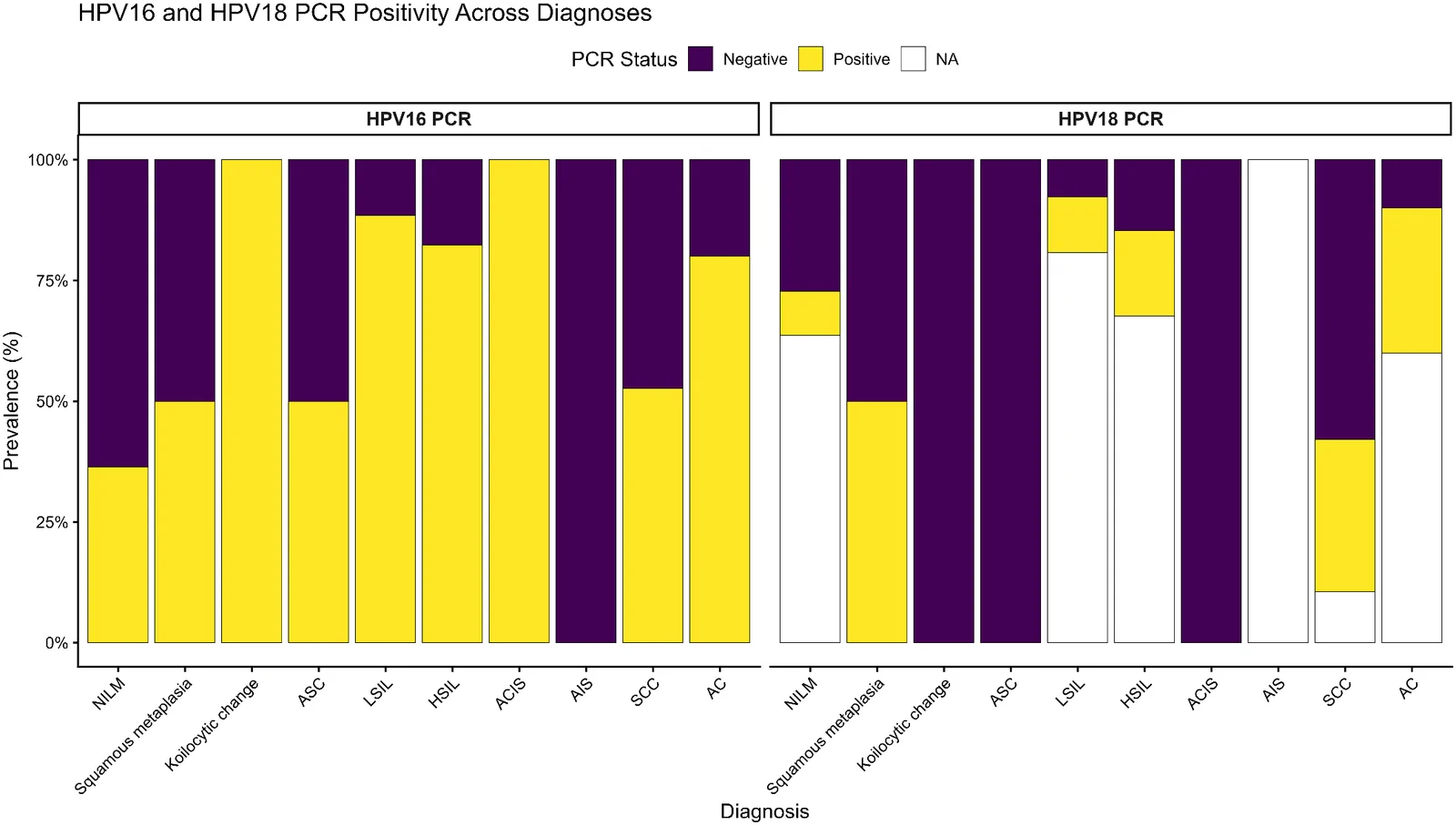
Distribution of HPV16 and HPV18 PCR status across diagnostic categories including NILM (negative for intraepithelial lesion or malignancy), squamous metaplasia, koilocytotic change, ASC-US/ASC-H (atypical squamous cells), LSIL (low-grade squamous intraepithelial lesion), HSIL (high-grade squamous intraepithelial lesion), AIS (adenocarcinoma *in situ*), SCC (squamous cell carcinoma), and adenocarcinoma (AC). Bars represent the proportion of PCR-negative, PCR-positive, and missing (NA) results within each diagnostic group.

The distribution of HPV16 and HPV18 PCR status across diagnostic categories is shown in **Figure 2**. Overall, HPV positivity increased progressively with disease severity for both HPV types. In HPV16, a higher proportion of positive cases was observed in low-grade lesions (LSIL) and further increased in high-grade lesions (HSIL) and invasive cancers (SCC and adenocarcinoma), while NILM and benign conditions showed a predominance of negative results. A similar but comparatively less pronounced pattern was observed for HPV18, where positivity was present across lesion categories but showed lower proportions in early-stage and benign diagnoses.

**Figure 3:**
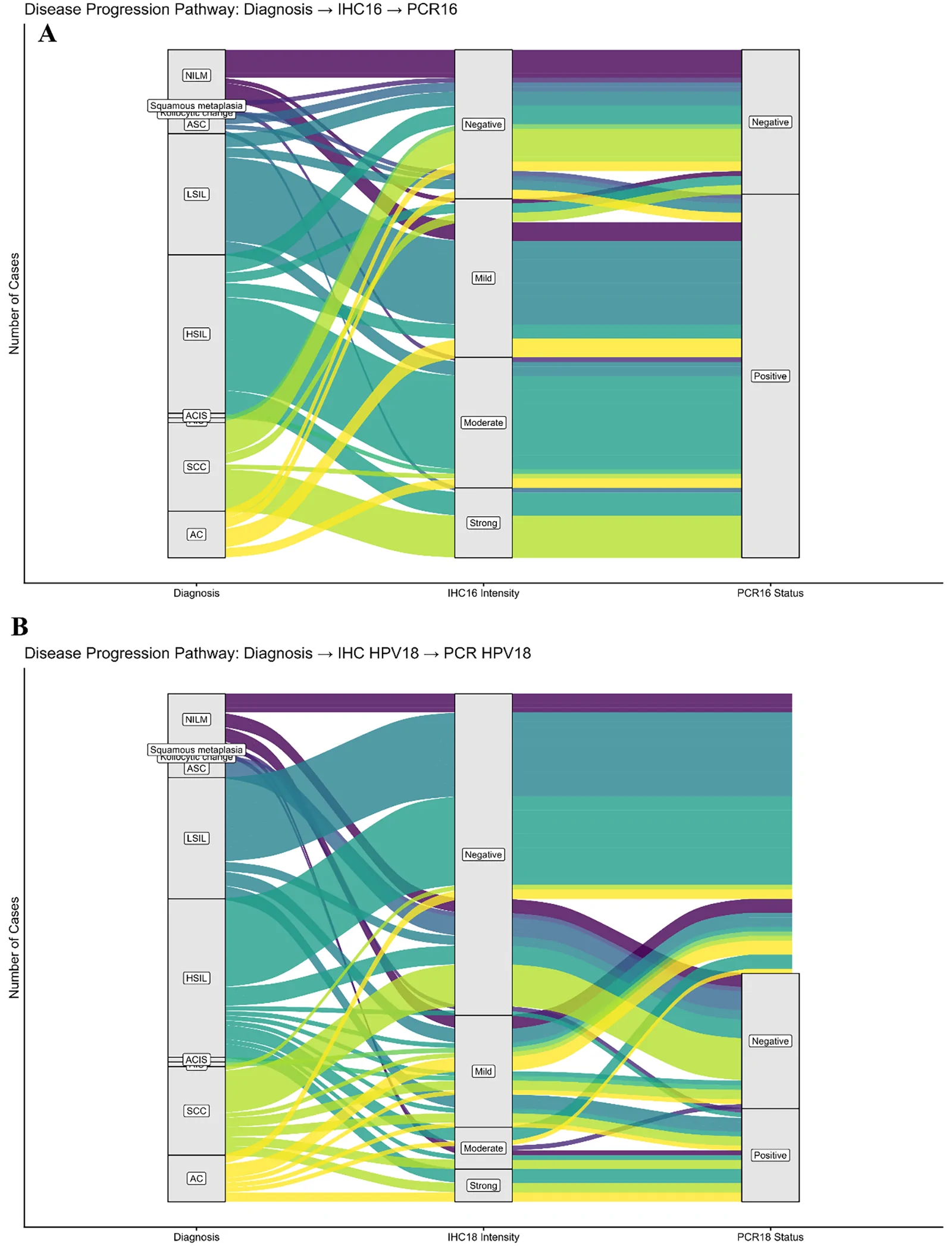
Disease progression pathways from histopathological diagnosis to IHC expression and PCR status for **(A)** HPV16 and **(B)** HPV18. Flow width represents the number of cases transitioning across diagnostic categories, IHC expression levels (negative, mild, moderate, and strong), and PCR outcomes (positive or negative). Higher-grade lesions generally demonstrated stronger IHC expression and greater PCR positivity for both HPV16 and HPV18.

Similarly, Sankey pathway analysis demonstrated a progressive relationship between histopathological diagnosis, IHC staining intensity, and PCR positivity for both HPV16 and HPV18. For HPV16, higher-grade lesions, particularly HSIL, SCC, and AC, predominantly transitioned to moderate-to-strong IHC expression and were largely associated with PCR-positive status, whereas NILM and low-grade lesions were more frequently linked to negative or mild IHC intensity and PCR-negative results. A similar trend was observed for HPV18, although a greater proportion of cases exhibited negative or mild IHC staining with PCR-negative outcomes, indicating comparatively lower positivity than HPV16 (**Figure 3**).

### Diagnostic performance of HPV16 and HPV18 biomarkers against PCR

Concordance analysis demonstrated substantial agreement between immunohistochemistry (IHC) and PCR findings for both HPV16 and HPV18. For HPV16, 72 cases were concordantly positive and 26 concordantly negative, whereas 11 discordant cases were identified, including 6 PCR-positive/IHC-negative and 5 PCR-negative/IHC-positive cases.

Similarly, For HPV18, 32 cases were concordantly positive and 64 concordantly negative, whereas 13 discordant cases were identified, comprising 5 PCR-positive/IHC-negative and 8 PCR-negative/IHC-positive cases (**Table 3**).

**Table 3:** Diagnostic performance of HPV16 and HPV18 biomarkers.

Marker	IHC positive definition	TP	TN	FP	FN	Sensitivity, % (95% CI)	Specificity, % (95% CI)	Accuracy, %	PPV, %	NPV, %
HPV16 E7	Mild/Moderate/Strong	72	26	5	6	92.3 (84.0–97.1)	83.9 (66.3–94.5)	89.9	93.5	81.2
HPV16 E7	Moderate/Strong	43	31	0	35	55.1 (43.4–66.4)	100.0 (88.8–100.0)	67.9	100.0	47.0
HPV18 E7	Mild/Moderate/Strong	32	64	8	5	86.5 (71.2–95.5)	88.9 (79.3–95.1)	88.1	80.0	92.8
HPV18 E7	Moderate/Strong	15	71	1	22	40.5 (24.8–57.9)	98.6 (92.5–100.0)	78.9	93.8	76.3
Marker	Sensitivity (95% CI)			Specificity (95% CI)	Accuracy (95% CI)			PPV (95% CI)		NPV (95% CI)
HPV16 E7	92.3 (84.0–97.1)			83.9 (66.3–94.5)	89.9 (82.7–94.9)			93.5 (85.5–97.9)		81.2 (63.6–92.8)
HPV18 E7	86.5 (71.2–95.5)			88.9 (79.3–95.1)	88.1 (80.5–93.5)			80.0 (64.4–90.9)		92.8 (83.9–97.6)

The diagnostic performance of HPV16 and HPV18 biomarkers is summarized in **Table 3**. Overall, both markers demonstrated good discriminatory ability for disease classification, with HPV16 consistently outperforming HPV18 across all evaluated metrics. HPV16 achieved a sensitivity of 92.3%, specificity of 83.9%, and an overall accuracy of 89.9%, corresponding to an area under the curve (AUC) of 0.925. In comparison, HPV18 showed slightly reduced performance, with a sensitivity of 90.0%, specificity of 79.3%, accuracy of 83.7%, and an AUC of 0.894.

These findings indicate that both HPV16 and HPV18 provide reliable diagnostic utility; however, HPV16 exhibits superior overall classification performance, particularly in terms of specificity and AUC, suggesting stronger discriminatory power between disease and non-disease states.

**Figure 4:**
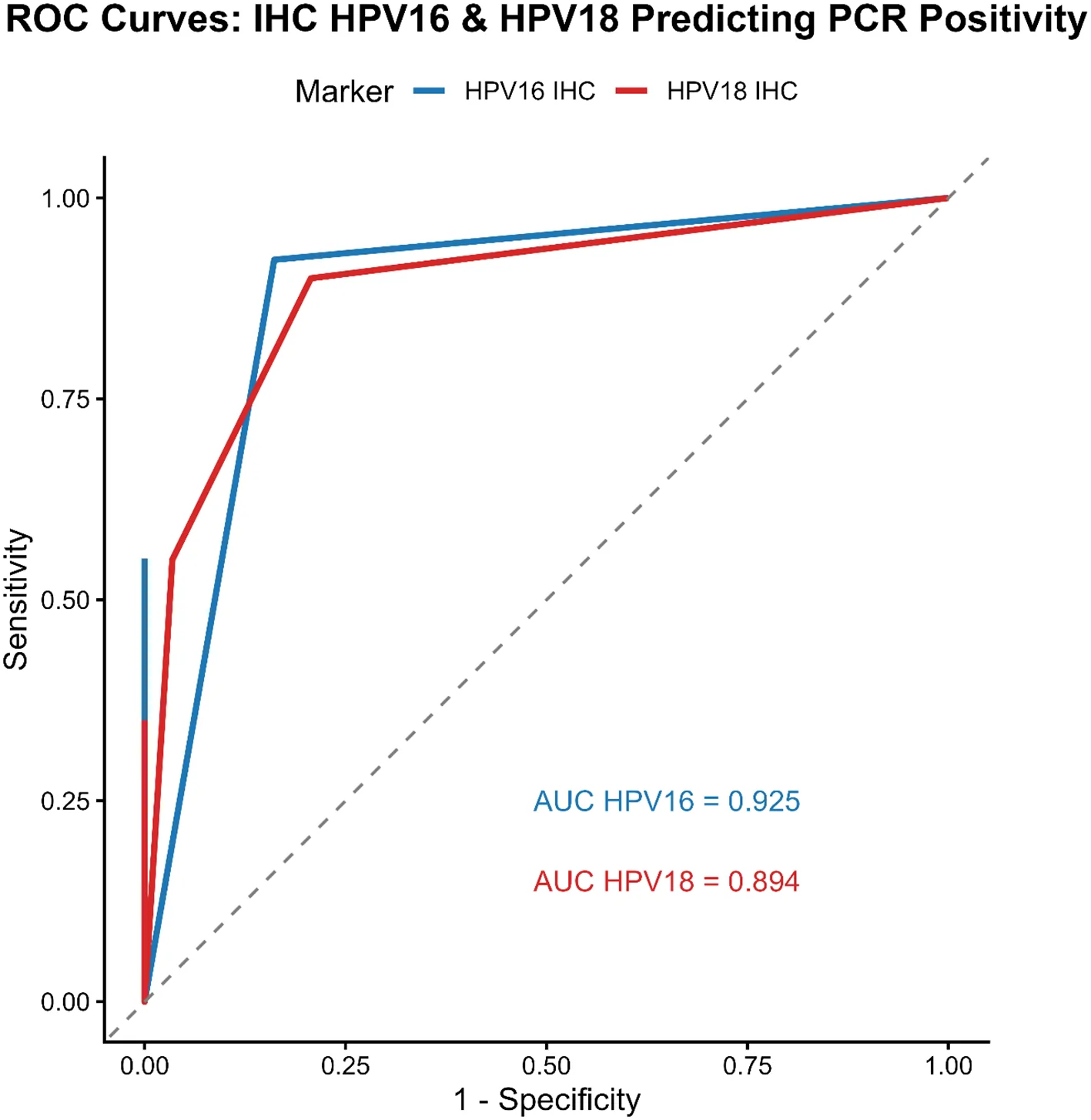
Receiver operating characteristic (ROC) curves of HPV16 and HPV18 IHC for predicting PCR positivity. Receiver operating characteristic (ROC) curves showing the diagnostic performance of immunohistochemistry (IHC) for predicting PCR positivity in HPV16 (blue) and HPV18 (red). HPV16 demonstrated a higher area under the curve (AUC = 0.925) compared to HPV18 (AUC = 0.894), indicating excellent predictive accuracy for both markers.

ROC curve analysis demonstrated strong predictive performance of IHC for PCR positivity as shown in **Figure 4**, with HPV16 showing slightly higher diagnostic accuracy (AUC = 0.925) than HPV18 (AUC = 0.894).

Sensitivity analysis using a more stringent IHC threshold demonstrated a substantial effect of the staining cutoff on diagnostic performance. When mild, moderate, and strong staining were considered positive, HPV16 E7 IHC showed a sensitivity of 92.3% (95% CI: 84.0–97.1) and specificity of 83.9% (95% CI: 66.3–94.5), whereas HPV18 E7 IHC showed a sensitivity of 86.5% (95% CI: 71.2–95.5) and specificity of 88.9% (95% CI: 79.3–95.1). When the positivity threshold was restricted to moderate or strong staining, sensitivity decreased to 55.1% (95% CI: 43.4–66.4) for HPV16 E7 and 40.5% (95% CI: 24.8–57.9) for HPV18 E7, while specificity increased to 100.0% (95% CI: 88.8–100.0) and 98.6% (95% CI: 92.5–100.0) as depicted in **Table 3**, respectively. These findings indicate that inclusion of mild staining substantially improved sensitivity at the expense of specificity.

### Multivariable logistic regression analysis of HPV16 and HPV18 models

Firth’s penalized logistic regression was used to address potential small-sample bias and separation in the multivariable models. After adjustment for age, HPV16 E7 IHC remained strongly associated with HPV16 PCR positivity (OR = 51.34, 95% CI: 16.57–191.23, P < 0.001). HPV18 E7 IHC also showed a strong association with HPV18 PCR positivity (OR = 45.34, 95% CI: 15.21–163.52, P < 0.001). Age was not independently associated with HPV16 PCR positivity (OR = 0.99, 95% CI: 0.93–1.05, P = 0.744) or HPV18 PCR positivity (OR = 0.98, 95% CI: 0.92–1.04, P = 0.487) (**Table 4**).

**Table 4:** Logistic regression analysis of predictors.

Model	Predictor	OR	95% CI	P-value
HPV16	HPV16 E7 IHC	51.34	16.57–191.23	<0.001
HPV16	Age	0.99	0.93–1.05	0.744
HPV18	HPV18 E7 IHC	45.34	15.21–163.52	<0.001
HPV18	Age	0.98	0.92–1.04	0.487

Odds ratios and 95% confidence intervals were estimated using Firth's penalized logistic regression to reduce bias associated with sparse data and potential separation.

### Immunohistochemical localization of biomarker expression across cervical lesion grades

**Figure 5:**
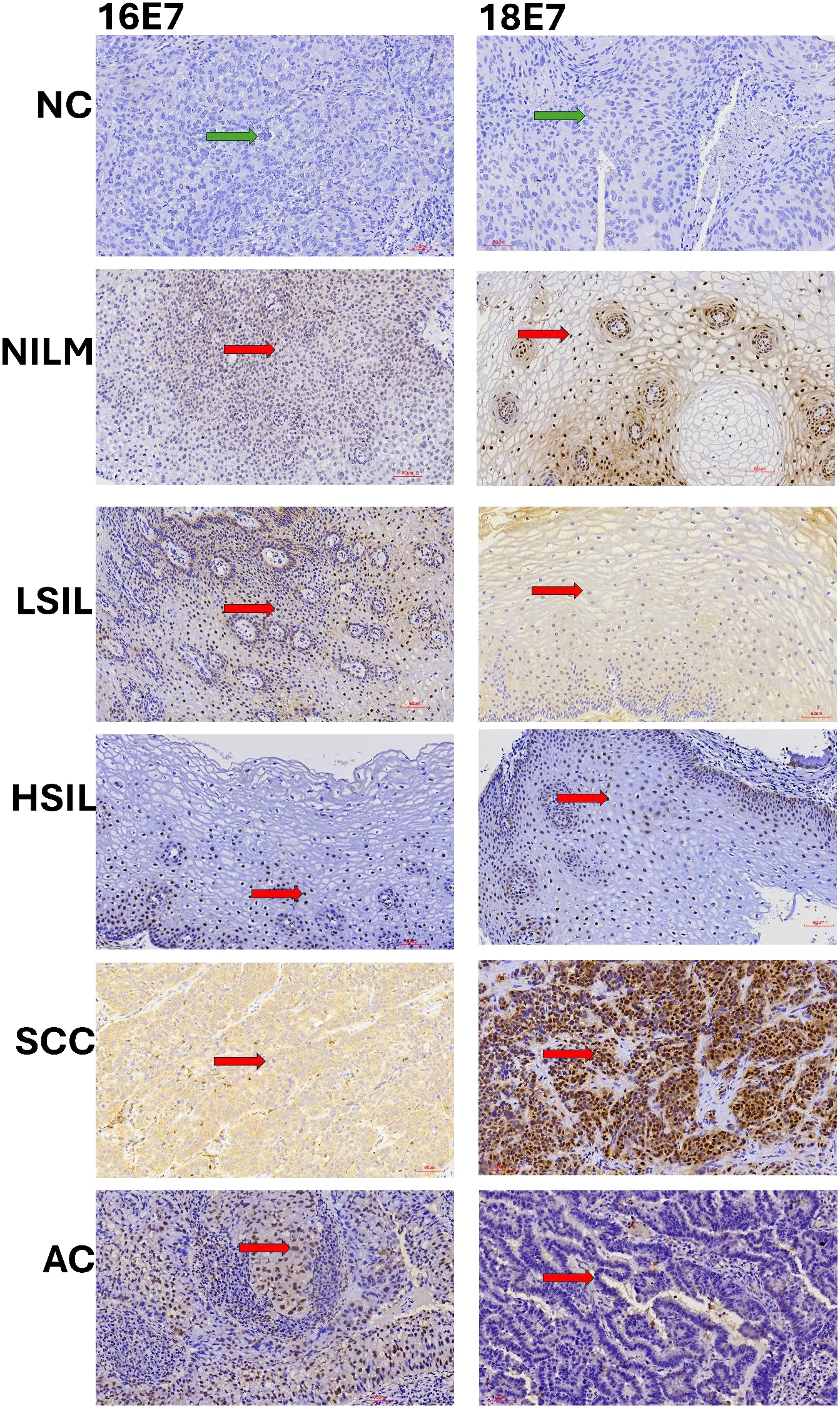
Representative immunohistochemical (IHC) micrographs of HPV 16 and 18 E7 IHC, demonstrating the localization and distribution of biomarker expression across different histopathological grades of cervical lesions. Negative control (NC) sections showed no specific immunoreactivity (green arrows). Low-grade lesions exhibited weak to mild nuclear staining mainly confined to the basal and parabasal epithelial layers. High-grade lesions and invasive carcinoma displayed strong and diffuse staining throughout the epithelial thickness (red arrows). All images were captured at ×20 magnification with a 60 µm scale bar.

Immunohistochemical (IHC) analysis demonstrated progressive changes in staining localization and intensity across the different histopathological grades (**Figure 5**). In the negative control (NC), no specific immunoreactivity was observed, confirming the specificity of the staining protocol. In low-grade lesions, weak to mild nuclear staining was predominantly localized within the basal and parabasal epithelial layers, with limited extension into the superficial layers. As the lesion grade progressed to moderate dysplasia, increased nuclear immunoreactivity was observed extending into the intermediate epithelial layers.

High-grade lesions and invasive carcinoma exhibited strong and diffuse nuclear staining throughout the full thickness of the epithelium and within malignant tumor nests. In some sections, cytoplasmic positivity was also evident alongside intense nuclear localization, particularly in areas showing keratin pearl formation and invasive cellular architecture.

## Discussion

The present study demonstrated that HPV16 and HPV18 immunohistochemical expression was strongly associated with increasing cervical lesion severity and showed substantial concordance with PCR findings. Overall, the findings support the potential utility of HPV E7-targeted IHC as a complementary tissue-based biomarker for evaluating cervical disease progression, particularly in higher-grade lesions and invasive carcinoma. Rather than functioning solely as a screening marker, the observed progressive increase in staining intensity and distribution across lesion grades suggests that E7 expression may reflect underlying oncogenic activity during cervical carcinogenesis.

The age-related increase observed across lesion severity categories is consistent with the established natural history of persistent high-risk HPV infection, where progression from low-grade lesions to invasive malignancy generally occurs over several years (24–26). Older age distributions among SCC and adenocarcinoma cases may therefore reflect cumulative viral persistence and prolonged molecular transformation. Similar age-associated progression patterns have been reported in previous cervical cancer studies, where invasive lesions were more commonly identified in older women compared with low-grade abnormalities (26–28). However, age was not an independent predictor in the multivariable models after adjustment for biomarker expression, suggesting that molecular activity may provide greater discriminatory value than demographic variables alone.

The high inter-observer agreement observed for both HPV16 E7 (κ = 0.880) and HPV18 E7 (κ = 0.912) supports the reproducibility of the categorical IHC scoring approach used in this study, although standardized scoring criteria and validation across independent pathology laboratories will be necessary before broader clinical implementation.

The progressive increase in HPV16E7 and HPV18E7 proteins immunoreactivity from benign lesions to HSIL and invasive carcinoma aligns with the recognized oncogenic roles of these high-risk HPV genotypes (5, 29). Notably, HPV16E7 demonstrated stronger diagnostic performance than HPV18E7 across sensitivity, specificity, accuracy, and AUC metrics. This observation is biologically plausible, as HPV16 is more frequently associated with squamous carcinogenesis and is often reported as the dominant genotype in cervical intraepithelial neoplasia and SCC (30). Previous studies have similarly shown higher prevalence and stronger biomarker performance for HPV16 compared with HPV18 in cervical lesions, particularly in squamous epithelial disease (3, 20).

The difference in performance between HPV16 and HPV18 E7 IHC may also reflect technical rather than purely biological factors. In particular, the two oncoproteins were detected using different commercially available antibodies, and differences in antibody affinity, epitope recognition, cross-reactivity, antigen preservation, and tissue accessibility may influence staining intensity and diagnostic sensitivity. Although both antibodies were applied using the same general staining workflow, their analytical characteristics were not independently validated against a common reference standard in the present study. Therefore, the apparent difference between HPV16 and HPV18 should not be attributed exclusively to differences in viral oncogenic biology.

Previous studies evaluating HPV E7 protein expression in cervical lesions have similarly suggested that tissue detection of viral oncoproteins may provide information related to biologically active HPV infection and lesion progression. The present findings extend this literature by evaluating HPV16 and HPV18 E7 expression concurrently across a broad spectrum of cervical pathology and by examining both staining localization and concordance with HPV DNA detection. The progressive transition observed in the present study from focal basal/parabasal staining in lower-grade lesions to more extensive and intense staining in HSIL and invasive carcinoma is broadly consistent with the concept that increasing E7 expression accompanies deregulated viral oncogene activity during cervical transformation. However, differences between studies in antibody specificity, scoring systems, specimen composition, HPV genotype distribution, and reference standards make direct comparison of diagnostic estimates difficult. The present results should therefore be interpreted as supportive of a complementary tissue-based role for E7 IHC rather than as evidence for replacing established molecular HPV testing.

A further consideration is the established role of p16INK4a immunohistochemistry in cervical pathology. Unlike HPV E7 IHC, p16 represents an indirect surrogate marker of transforming HPV activity through disruption of the pRb pathway and subsequent p16 overexpression. p16 IHC is already widely available and has established applications in the interpretation of morphologically equivocal cervical lesions. HPV E7 IHC offers a different biological readout by directly detecting a viral oncoprotein, and its tissue localization may therefore provide complementary information. However, the present study was not designed as a head-to-head comparison between E7 IHC and p16 IHC, and p16 staining was not performed in the study cohort. Consequently, no direct conclusion regarding the superiority of either marker can be made from the present data. Future prospective studies should compare HPV E7 IHC directly with p16 IHC, HPV molecular testing, and combined p16/HPV-based triage strategies using standardized clinical endpoints.

The substantial concordance between IHC and PCR findings further supports the diagnostic relevance of HPV E7 protein expression. Although PCR remains highly sensitive for viral nucleic acid detection, tissue-based IHC provides additional spatial and morphological information that cannot be obtained through molecular testing alone. The localization patterns observed in this study, particularly the transition from basal-restricted staining in low-grade lesions to diffuse full-thickness staining in HSIL and invasive carcinoma, are consistent with the progressive deregulation of viral oncogene expression during malignant transformation.

Importantly, the logistic regression analysis identified HPV16E7 and HPV18E7 IHC expression as independent predictors of disease status, even after adjustment for age and diagnostic score. The markedly higher odds ratio observed for HPV16 suggests an association with disease progression and may reflect differences in viral oncogenic potential between the two genotypes. Nevertheless, the wide confidence intervals indicate some degree of variability, likely related to sample size and subgroup distribution, and therefore these findings should be interpreted cautiously.

It is important to emphasize that HPV E7 immunohistochemistry should not be interpreted as a substitute for validated primary cervical cancer screening approaches based on HPV nucleic-acid testing. Rather, its potential clinical value lies in selected tissue-based diagnostic and confirmatory settings. Because E7 is a viral oncoprotein directly involved in HPV-mediated cellular transformation, its detection within morphologically abnormal epithelium may provide spatial information regarding biologically active HPV infection that cannot be obtained from nucleic-acid detection alone. Accordingly, E7 IHC may be particularly useful as a complementary biomarker in morphologically equivocal cervical lesions, in selected triage or confirmatory assessments, and in cases in which tissue localization of viral protein expression could assist pathological interpretation. These potential applications remain investigational and require prospective clinical validation before incorporation into routine screening algorithms.

From a diagnostic perspective, HPV E7 immunohistochemistry should be considered a complementary tissue-based biomarker rather than a replacement for primary HPV molecular screening. Its potential clinical value may be greatest in selected secondary or confirmatory settings, particularly when histomorphological findings are equivocal, when there is diagnostic uncertainty between reactive/metaplastic and HPV-associated dysplastic change, or when tissue-based confirmation of biologically active HPV-associated disease is desirable. The ability of E7 IHC to demonstrate the spatial distribution and intensity of viral oncoprotein expression within the lesional epithelium may provide information that is not available from HPV DNA detection alone. In this context, E7 IHC could potentially complement histopathological assessment and molecular HPV testing in diagnostically challenging cervical biopsies, rather than serve as a population-level screening assay. Such applications would require prospective validation against clinically relevant endpoints and direct comparison with established triage approaches before clinical adoption.

An important consideration is the relationship between HPV E7 IHC and p16INK4a immunohistochemistry, which is already widely used in cervical pathology as a surrogate marker of transforming HPV infection. p16 overexpression reflects disruption of the pRb pathway resulting from oncogenic HPV activity, whereas E7 IHC provides direct detection of a viral oncoprotein within the tissue. Thus, the two markers represent related but biologically distinct readouts of HPV-associated transformation. E7 IHC may therefore provide complementary information regarding the localization of viral protein expression, but the present study was not designed as a formal head-to-head comparison of E7 and p16 using an independent clinical reference standard. Future studies should directly compare both biomarkers using standardized pathology endpoints and clinically relevant outcomes.

The threshold used to define IHC positivity also influenced the diagnostic characteristics of E7 staining. Inclusion of mild staining as a positive result produced substantially higher sensitivity for both HPV16 E7 and HPV18 E7, whereas restricting positivity to moderate-to-strong staining markedly increased specificity but resulted in a considerable loss of sensitivity. This finding suggests that mild E7 expression may capture additional HPV-associated lesions that would be missed by a more stringent threshold, although some mild or focal staining may represent non-transforming viral infection or nonspecific/background staining. Therefore, the interpretation of mild E7 staining requires careful morphological correlation and should not be considered equivalent to diffuse moderate-to-strong staining. Importantly, the present sensitivity analysis demonstrates that the choice of threshold has a major influence on diagnostic performance and should be standardized in future prospective studies.

From a feasibility perspective, E7 IHC requires tissue biopsy or excision, formalin fixation, paraffin processing, antigen retrieval, antibody incubation, chromogenic detection, and microscopic interpretation. These requirements make it substantially less suitable for population-based screening than HPV molecular assays, which can be performed on non-invasive or minimally invasive specimens and are amenable to standardized high-throughput workflows. Nevertheless, in pathology laboratories where tissue sections and IHC infrastructure are already available, E7 IHC may be feasible as a targeted adjunctive test in selected diagnostically challenging cases. The present study did not perform a formal health-economic analysis; therefore, comparative cost-effectiveness cannot be inferred and should be evaluated in future implementation studies.

The present study has several strengths, including the integration of histopathological evaluation, HPV PCR comparison, immunohistochemical localization, and multivariable diagnostic analysis within a single cohort encompassing the spectrum of cervical lesion progression. Furthermore, assessment of tissue localization patterns provided additional pathological context regarding the biological distribution of HPV16 and HPV18 E7 expression during disease progression.

### Limitations

Some limitations should be considered when interpreting the findings of the present study. First, this investigation was conducted within a single-center retrospective cohort, which may limit the generalizability of the findings across broader and more diverse populations. External multicenter validation is therefore warranted.

Second, some histopathological categories, particularly glandular lesions such as adenocarcinoma *in situ* and adenocarcinoma, were represented by relatively small case numbers, potentially limiting statistical power for subgroup-specific interpretation.

Third, the study focused exclusively on HPV16 and HPV18 E7 expression. Although these genotypes are major contributors to cervical carcinogenesis, other high-risk HPV types also contribute substantially to cervical precancer and cancer, and their relative contribution varies geographically. Consequently, HPV16/18 E7 IHC alone would not identify all HPV-associated lesions, particularly in populations in which other oncogenic genotypes are prevalent. Broader genotype-specific E7 panels will therefore be necessary to determine whether the tissue-based approach can be generalized across different epidemiological settings.

Although PCR was used as the molecular reference method, discordant cases between PCR and immunohistochemistry were observed. These discrepancies may reflect differences in viral transcriptional activity, tissue heterogeneity, antigen preservation, viral load, sampling variability, or technical factors influencing immunohistochemical interpretation.

Additionally, while standardized scoring criteria and independent assessment were applied, immunohistochemical evaluation remains partially observer-dependent and may be subject to interobserver variability. An additional limitation is that HPV E7 IHC cannot currently be considered a primary cervical cancer screening test. Unlike validated HPV molecular assays, E7 IHC requires tissue sampling, histological processing, antigen retrieval, antibody-based detection, and microscopic interpretation. Its clinical performance may also vary according to antibody characteristics, staining protocols, scoring thresholds, and HPV genotype coverage. Therefore, the present findings should be interpreted as evidence for a potential complementary tissue-based application rather than as evidence supporting replacement of molecular HPV screening. Prospective studies comparing E7 IHC directly with validated screening and triage strategies are required before clinical implementation.

An additional limitation is that HPV E7 IHC cannot currently be considered a primary cervical cancer screening test. Unlike validated HPV molecular assays, E7 IHC requires tissue sampling, histological processing, antigen retrieval, antibody-based detection, and microscopic interpretation. Its clinical performance may also vary according to antibody characteristics, staining protocols, scoring thresholds, and HPV genotype coverage. Therefore, the present findings should be interpreted as evidence for a potential complementary tissue-based application rather than as evidence supporting replacement of molecular HPV screening. Prospective studies comparing E7 IHC directly with validated screening and triage strategies are required before clinical implementation.

The diagnostic estimates in this study were derived from comparison with HPV DNA PCR and should not be interpreted as clinical sensitivity or specificity against longitudinal clinical endpoints. Clinical sensitivity and specificity would ideally be evaluated against clinically relevant outcomes such as histologically confirmed disease requiring treatment, persistent high-grade disease, progression, or clinically meaningful disease over follow-up. Such outcomes were not available in this retrospective archival cohort. Prospective studies incorporating longitudinal follow-up are therefore required to establish the clinical sensitivity, specificity, and prognostic value of HPV E7 IHC.

Although PCR-negative thyroid tissue and reagent-negative controls were included, an HPV-negative normal cervical tissue control would have provided a more tissue-matched assessment of nonspecific staining within cervical squamous epithelium. The use of thyroid tissue as the negative tissue control is therefore acknowledged as a methodological limitation.

Finally, the retrospective cross-sectional design limits assessment of temporal and prognostic relationships between HPV E7 expression and long-term clinical outcomes such as lesion persistence, progression, recurrence, or treatment response. Longitudinal prospective studies are therefore required to establish the prognostic significance of HPV E7 immunohistochemistry in cervical disease.

## Conclusion

The present study demonstrated that HPV16 and HPV18 E7 immunohistochemical expression increases progressively with cervical lesion severity and exhibits substantial concordance with HPV DNA PCR findings. Both biomarkers demonstrated favorable diagnostic performance, with HPV16 showing comparatively stronger discriminatory ability than HPV18.

The observed transition from focal basal/parabasal staining in low-grade lesions to diffuse full-thickness epithelial and tumor-associated expression in high-grade lesions and invasive carcinoma further supports the biological relevance of HPV E7 activity during cervical carcinogenesis.

Collectively, these findings support the potential role of HPV16 and HPV18 E7 immunohistochemistry as complementary tissue-based biomarkers in cervical pathology. Integration of E7 immunohistochemistry with routine histopathological evaluation and molecular HPV testing may enhance lesion characterization and diagnostic confidence, particularly in morphologically equivocal or diagnostically challenging cases.

Further multicenter studies with larger cohorts and longitudinal follow-up are warranted to validate the diagnostic and potential prognostic utility of HPV E7 immunohistochemistry in HPV-associated cervical disease.

## Data Availability

The original contributions presented in the study are included in the article/**Supplementary Material**. Further inquiries can be directed to the corresponding author.
