# Supplementary material for: Diagnostic utility and tissue localization of HPV16 and HPV18 E7 immunohistochemistry in cervical lesion progression: a comparative study with PCR
Source: Front Oncol. 2026 Sep 10;16:1942646. doi: 10.3389/fonc.2026.1942646 (PMC13600906; doi:10.3389/fonc.2026.1942646)
Supplement: Supplementary file 1 [file SupplementaryFile1.docx]

**Figure S1.** Heatmap correlation matrix illustrating the relationships among age, diagnosis score, HPV16, HPV18, p16, and p18 biomarkers. Positive correlations are represented by blue shades, while negative correlations are indicated by red shades. The intensity of the color corresponds to the strength of the correlation coefficient. Strong positive associations were observed between IHC and PCR of HPV16 and 18, suggesting coordinated biomarker expression patterns associated with HPV-related disease progression.

| **Characteristic** | **NILM**  N = 11^1^ | **Squamous metaplasia**  N = 2^1^ | **Koilocytic change**  N = 1^1^ | **ASC**  N = 4^1^ | **LSIL**  N = 26^1^ | **HSIL**  N = 34^1^ | **ACIS**  N = 1^1^ | **AIS**  N = 1^1^ | **SCC**  N = 19^1^ | **AC**  N = 10^1^ |
| --- | --- | --- | --- | --- | --- | --- | --- | --- | --- | --- |
| age | 41 ± 7 | 33 ± 2 | 33 ± NA | 42 ± 6 | 42 ± 6 | 54 ± 5 | 43 ± NA | 47 ± NA | 63 ± 2 | 60 ± 5 |
|  |  |  |  |  |  |  |  |  |  |  |
|  | 1 (9.1%) | 0 (0%) | 0 (0%) | 0 (0%) | 0 (0%) | 0 (0%) | 0 (0%) | 0 (0%) | 0 (0%) | 0 (0%) |
|  | 1 (9.1%) | 0 (0%) | 0 (0%) | 0 (0%) | 0 (0%) | 0 (0%) | 0 (0%) | 0 (0%) | 0 (0%) | 0 (0%) |
|  | 0 (0%) | 0 (0%) | 0 (0%) | 0 (0%) | 0 (0%) | 1 (2.9%) | 0 (0%) | 0 (0%) | 0 (0%) | 0 (0%) |
|  | 0 (0%) | 0 (0%) | 0 (0%) | 0 (0%) | 1 (3.8%) | 0 (0%) | 0 (0%) | 0 (0%) | 0 (0%) | 0 (0%) |
|  | 0 (0%) | 0 (0%) | 0 (0%) | 0 (0%) | 0 (0%) | 1 (2.9%) | 0 (0%) | 0 (0%) | 0 (0%) | 0 (0%) |
|  | 0 (0%) | 0 (0%) | 0 (0%) | 0 (0%) | 0 (0%) | 1 (2.9%) | 0 (0%) | 0 (0%) | 0 (0%) | 0 (0%) |
|  | 0 (0%) | 0 (0%) | 0 (0%) | 0 (0%) | 0 (0%) | 1 (2.9%) | 0 (0%) | 0 (0%) | 0 (0%) | 0 (0%) |
|  | 0 (0%) | 0 (0%) | 0 (0%) | 0 (0%) | 0 (0%) | 1 (2.9%) | 0 (0%) | 0 (0%) | 0 (0%) | 0 (0%) |
|  | 0 (0%) | 0 (0%) | 0 (0%) | 0 (0%) | 0 (0%) | 0 (0%) | 0 (0%) | 0 (0%) | 0 (0%) | 1 (10%) |
|  | 0 (0%) | 0 (0%) | 0 (0%) | 0 (0%) | 0 (0%) | 1 (2.9%) | 0 (0%) | 0 (0%) | 0 (0%) | 0 (0%) |
|  | 0 (0%) | 0 (0%) | 0 (0%) | 0 (0%) | 0 (0%) | 1 (2.9%) | 0 (0%) | 0 (0%) | 0 (0%) | 0 (0%) |
|  | 0 (0%) | 0 (0%) | 0 (0%) | 0 (0%) | 0 (0%) | 1 (2.9%) | 0 (0%) | 0 (0%) | 0 (0%) | 0 (0%) |
|  | 1 (9.1%) | 0 (0%) | 0 (0%) | 0 (0%) | 0 (0%) | 0 (0%) | 0 (0%) | 0 (0%) | 0 (0%) | 0 (0%) |
|  | 0 (0%) | 0 (0%) | 0 (0%) | 0 (0%) | 0 (0%) | 0 (0%) | 0 (0%) | 0 (0%) | 0 (0%) | 1 (10%) |
|  | 0 (0%) | 0 (0%) | 0 (0%) | 0 (0%) | 1 (3.8%) | 0 (0%) | 0 (0%) | 0 (0%) | 0 (0%) | 0 (0%) |
|  | 1 (9.1%) | 0 (0%) | 0 (0%) | 0 (0%) | 0 (0%) | 0 (0%) | 0 (0%) | 0 (0%) | 0 (0%) | 0 (0%) |
|  | 0 (0%) | 0 (0%) | 0 (0%) | 0 (0%) | 0 (0%) | 1 (2.9%) | 0 (0%) | 0 (0%) | 0 (0%) | 0 (0%) |
|  | 0 (0%) | 0 (0%) | 0 (0%) | 0 (0%) | 1 (3.8%) | 0 (0%) | 0 (0%) | 0 (0%) | 0 (0%) | 0 (0%) |
|  | 0 (0%) | 0 (0%) | 0 (0%) | 0 (0%) | 1 (3.8%) | 0 (0%) | 0 (0%) | 0 (0%) | 0 (0%) | 0 (0%) |
|  | 0 (0%) | 0 (0%) | 0 (0%) | 0 (0%) | 0 (0%) | 1 (2.9%) | 0 (0%) | 0 (0%) | 0 (0%) | 0 (0%) |
|  | 0 (0%) | 0 (0%) | 0 (0%) | 0 (0%) | 0 (0%) | 1 (2.9%) | 0 (0%) | 0 (0%) | 0 (0%) | 0 (0%) |
|  | 0 (0%) | 0 (0%) | 0 (0%) | 0 (0%) | 0 (0%) | 1 (2.9%) | 0 (0%) | 0 (0%) | 0 (0%) | 0 (0%) |
|  | 0 (0%) | 0 (0%) | 0 (0%) | 0 (0%) | 1 (3.8%) | 0 (0%) | 0 (0%) | 0 (0%) | 0 (0%) | 0 (0%) |
|  | 0 (0%) | 0 (0%) | 0 (0%) | 0 (0%) | 0 (0%) | 1 (2.9%) | 0 (0%) | 0 (0%) | 0 (0%) | 0 (0%) |
|  | 0 (0%) | 0 (0%) | 0 (0%) | 0 (0%) | 1 (3.8%) | 0 (0%) | 0 (0%) | 0 (0%) | 0 (0%) | 0 (0%) |
|  | 1 (9.1%) | 0 (0%) | 0 (0%) | 0 (0%) | 0 (0%) | 0 (0%) | 0 (0%) | 0 (0%) | 0 (0%) | 0 (0%) |
|  | 0 (0%) | 0 (0%) | 0 (0%) | 0 (0%) | 1 (3.8%) | 0 (0%) | 0 (0%) | 0 (0%) | 0 (0%) | 0 (0%) |
|  | 0 (0%) | 0 (0%) | 0 (0%) | 0 (0%) | 1 (3.8%) | 0 (0%) | 0 (0%) | 0 (0%) | 0 (0%) | 0 (0%) |
|  | 0 (0%) | 0 (0%) | 0 (0%) | 0 (0%) | 0 (0%) | 1 (2.9%) | 0 (0%) | 0 (0%) | 0 (0%) | 0 (0%) |
|  | 0 (0%) | 0 (0%) | 0 (0%) | 0 (0%) | 0 (0%) | 1 (2.9%) | 0 (0%) | 0 (0%) | 0 (0%) | 0 (0%) |
|  | 0 (0%) | 0 (0%) | 0 (0%) | 0 (0%) | 0 (0%) | 0 (0%) | 0 (0%) | 0 (0%) | 0 (0%) | 1 (10%) |
|  | 0 (0%) | 0 (0%) | 0 (0%) | 0 (0%) | 1 (3.8%) | 0 (0%) | 0 (0%) | 0 (0%) | 0 (0%) | 0 (0%) |
|  | 0 (0%) | 0 (0%) | 0 (0%) | 0 (0%) | 1 (3.8%) | 0 (0%) | 0 (0%) | 0 (0%) | 0 (0%) | 0 (0%) |
|  | 0 (0%) | 0 (0%) | 0 (0%) | 0 (0%) | 1 (3.8%) | 0 (0%) | 0 (0%) | 0 (0%) | 0 (0%) | 0 (0%) |
|  | 1 (9.1%) | 0 (0%) | 0 (0%) | 0 (0%) | 0 (0%) | 0 (0%) | 0 (0%) | 0 (0%) | 0 (0%) | 0 (0%) |
|  | 0 (0%) | 0 (0%) | 0 (0%) | 0 (0%) | 1 (3.8%) | 0 (0%) | 0 (0%) | 0 (0%) | 0 (0%) | 0 (0%) |
|  | 0 (0%) | 0 (0%) | 0 (0%) | 0 (0%) | 0 (0%) | 0 (0%) | 0 (0%) | 0 (0%) | 0 (0%) | 1 (10%) |
|  | 0 (0%) | 0 (0%) | 0 (0%) | 0 (0%) | 0 (0%) | 0 (0%) | 0 (0%) | 1 (100%) | 0 (0%) | 0 (0%) |
|  | 0 (0%) | 0 (0%) | 0 (0%) | 0 (0%) | 0 (0%) | 1 (2.9%) | 0 (0%) | 0 (0%) | 0 (0%) | 0 (0%) |
|  | 0 (0%) | 0 (0%) | 0 (0%) | 0 (0%) | 0 (0%) | 0 (0%) | 0 (0%) | 0 (0%) | 0 (0%) | 1 (10%) |
|  | 0 (0%) | 1 (50%) | 0 (0%) | 0 (0%) | 0 (0%) | 0 (0%) | 0 (0%) | 0 (0%) | 0 (0%) | 0 (0%) |
|  | 0 (0%) | 0 (0%) | 0 (0%) | 0 (0%) | 1 (3.8%) | 0 (0%) | 0 (0%) | 0 (0%) | 0 (0%) | 0 (0%) |
|  | 0 (0%) | 0 (0%) | 0 (0%) | 0 (0%) | 0 (0%) | 0 (0%) | 0 (0%) | 0 (0%) | 1 (5.3%) | 0 (0%) |
|  | 0 (0%) | 0 (0%) | 0 (0%) | 0 (0%) | 0 (0%) | 0 (0%) | 0 (0%) | 0 (0%) | 1 (5.3%) | 0 (0%) |
|  | 0 (0%) | 0 (0%) | 0 (0%) | 1 (25%) | 0 (0%) | 0 (0%) | 0 (0%) | 0 (0%) | 0 (0%) | 0 (0%) |
|  | 0 (0%) | 0 (0%) | 0 (0%) | 0 (0%) | 0 (0%) | 1 (2.9%) | 0 (0%) | 0 (0%) | 0 (0%) | 0 (0%) |
|  | 0 (0%) | 0 (0%) | 0 (0%) | 0 (0%) | 0 (0%) | 1 (2.9%) | 0 (0%) | 0 (0%) | 0 (0%) | 0 (0%) |
|  | 0 (0%) | 0 (0%) | 0 (0%) | 0 (0%) | 0 (0%) | 0 (0%) | 1 (100%) | 0 (0%) | 0 (0%) | 0 (0%) |
|  | 0 (0%) | 0 (0%) | 0 (0%) | 0 (0%) | 0 (0%) | 0 (0%) | 0 (0%) | 0 (0%) | 1 (5.3%) | 0 (0%) |
|  | 0 (0%) | 0 (0%) | 0 (0%) | 0 (0%) | 0 (0%) | 0 (0%) | 0 (0%) | 0 (0%) | 1 (5.3%) | 0 (0%) |
|  | 0 (0%) | 0 (0%) | 0 (0%) | 0 (0%) | 0 (0%) | 1 (2.9%) | 0 (0%) | 0 (0%) | 0 (0%) | 0 (0%) |
|  | 0 (0%) | 0 (0%) | 0 (0%) | 0 (0%) | 0 (0%) | 0 (0%) | 0 (0%) | 0 (0%) | 1 (5.3%) | 0 (0%) |
|  | 0 (0%) | 0 (0%) | 0 (0%) | 0 (0%) | 0 (0%) | 0 (0%) | 0 (0%) | 0 (0%) | 0 (0%) | 1 (10%) |
|  | 0 (0%) | 0 (0%) | 0 (0%) | 0 (0%) | 0 (0%) | 0 (0%) | 0 (0%) | 0 (0%) | 1 (5.3%) | 0 (0%) |
|  | 0 (0%) | 0 (0%) | 0 (0%) | 0 (0%) | 0 (0%) | 1 (2.9%) | 0 (0%) | 0 (0%) | 0 (0%) | 0 (0%) |
|  | 0 (0%) | 0 (0%) | 0 (0%) | 0 (0%) | 0 (0%) | 1 (2.9%) | 0 (0%) | 0 (0%) | 0 (0%) | 0 (0%) |
|  | 0 (0%) | 0 (0%) | 0 (0%) | 0 (0%) | 0 (0%) | 0 (0%) | 0 (0%) | 0 (0%) | 0 (0%) | 1 (10%) |
|  | 0 (0%) | 0 (0%) | 0 (0%) | 0 (0%) | 0 (0%) | 0 (0%) | 0 (0%) | 0 (0%) | 1 (5.3%) | 0 (0%) |
|  | 0 (0%) | 0 (0%) | 0 (0%) | 0 (0%) | 0 (0%) | 0 (0%) | 0 (0%) | 0 (0%) | 1 (5.3%) | 0 (0%) |
|  | 0 (0%) | 0 (0%) | 0 (0%) | 0 (0%) | 0 (0%) | 0 (0%) | 0 (0%) | 0 (0%) | 1 (5.3%) | 0 (0%) |
|  | 0 (0%) | 0 (0%) | 0 (0%) | 0 (0%) | 0 (0%) | 1 (2.9%) | 0 (0%) | 0 (0%) | 0 (0%) | 0 (0%) |
|  | 0 (0%) | 0 (0%) | 0 (0%) | 0 (0%) | 1 (3.8%) | 0 (0%) | 0 (0%) | 0 (0%) | 0 (0%) | 0 (0%) |
|  | 0 (0%) | 0 (0%) | 0 (0%) | 0 (0%) | 0 (0%) | 1 (2.9%) | 0 (0%) | 0 (0%) | 0 (0%) | 0 (0%) |
|  | 0 (0%) | 0 (0%) | 0 (0%) | 0 (0%) | 1 (3.8%) | 0 (0%) | 0 (0%) | 0 (0%) | 0 (0%) | 0 (0%) |
|  | 0 (0%) | 0 (0%) | 0 (0%) | 1 (25%) | 0 (0%) | 0 (0%) | 0 (0%) | 0 (0%) | 0 (0%) | 0 (0%) |
|  | 0 (0%) | 0 (0%) | 0 (0%) | 0 (0%) | 1 (3.8%) | 0 (0%) | 0 (0%) | 0 (0%) | 0 (0%) | 0 (0%) |
|  | 0 (0%) | 0 (0%) | 0 (0%) | 1 (25%) | 0 (0%) | 0 (0%) | 0 (0%) | 0 (0%) | 0 (0%) | 0 (0%) |
|  | 0 (0%) | 0 (0%) | 0 (0%) | 0 (0%) | 0 (0%) | 0 (0%) | 0 (0%) | 0 (0%) | 1 (5.3%) | 0 (0%) |
|  | 0 (0%) | 0 (0%) | 0 (0%) | 0 (0%) | 0 (0%) | 1 (2.9%) | 0 (0%) | 0 (0%) | 0 (0%) | 0 (0%) |
|  | 0 (0%) | 0 (0%) | 0 (0%) | 0 (0%) | 0 (0%) | 0 (0%) | 0 (0%) | 0 (0%) | 0 (0%) | 1 (10%) |
|  | 0 (0%) | 0 (0%) | 0 (0%) | 0 (0%) | 0 (0%) | 1 (2.9%) | 0 (0%) | 0 (0%) | 0 (0%) | 0 (0%) |
|  | 1 (9.1%) | 0 (0%) | 0 (0%) | 0 (0%) | 0 (0%) | 0 (0%) | 0 (0%) | 0 (0%) | 0 (0%) | 0 (0%) |
|  | 1 (9.1%) | 0 (0%) | 0 (0%) | 0 (0%) | 0 (0%) | 0 (0%) | 0 (0%) | 0 (0%) | 0 (0%) | 0 (0%) |
|  | 0 (0%) | 0 (0%) | 0 (0%) | 0 (0%) | 0 (0%) | 0 (0%) | 0 (0%) | 0 (0%) | 1 (5.3%) | 0 (0%) |
|  | 0 (0%) | 0 (0%) | 0 (0%) | 0 (0%) | 0 (0%) | 0 (0%) | 0 (0%) | 0 (0%) | 1 (5.3%) | 0 (0%) |
|  | 0 (0%) | 0 (0%) | 0 (0%) | 0 (0%) | 0 (0%) | 1 (2.9%) | 0 (0%) | 0 (0%) | 0 (0%) | 0 (0%) |
|  | 0 (0%) | 0 (0%) | 0 (0%) | 1 (25%) | 0 (0%) | 0 (0%) | 0 (0%) | 0 (0%) | 0 (0%) | 0 (0%) |
|  | 0 (0%) | 0 (0%) | 0 (0%) | 0 (0%) | 0 (0%) | 0 (0%) | 0 (0%) | 0 (0%) | 1 (5.3%) | 0 (0%) |
|  | 0 (0%) | 0 (0%) | 0 (0%) | 0 (0%) | 0 (0%) | 0 (0%) | 0 (0%) | 0 (0%) | 1 (5.3%) | 0 (0%) |
|  | 0 (0%) | 0 (0%) | 0 (0%) | 0 (0%) | 1 (3.8%) | 0 (0%) | 0 (0%) | 0 (0%) | 0 (0%) | 0 (0%) |
|  | 0 (0%) | 0 (0%) | 0 (0%) | 0 (0%) | 0 (0%) | 0 (0%) | 0 (0%) | 0 (0%) | 1 (5.3%) | 0 (0%) |
|  | 0 (0%) | 0 (0%) | 0 (0%) | 0 (0%) | 0 (0%) | 0 (0%) | 0 (0%) | 0 (0%) | 1 (5.3%) | 0 (0%) |
|  | 0 (0%) | 0 (0%) | 1 (100%) | 0 (0%) | 0 (0%) | 0 (0%) | 0 (0%) | 0 (0%) | 0 (0%) | 0 (0%) |
|  | 1 (9.1%) | 0 (0%) | 0 (0%) | 0 (0%) | 0 (0%) | 0 (0%) | 0 (0%) | 0 (0%) | 0 (0%) | 0 (0%) |
|  | 0 (0%) | 1 (50%) | 0 (0%) | 0 (0%) | 0 (0%) | 0 (0%) | 0 (0%) | 0 (0%) | 0 (0%) | 0 (0%) |
|  | 1 (9.1%) | 0 (0%) | 0 (0%) | 0 (0%) | 0 (0%) | 0 (0%) | 0 (0%) | 0 (0%) | 0 (0%) | 0 (0%) |
|  | 0 (0%) | 0 (0%) | 0 (0%) | 0 (0%) | 0 (0%) | 1 (2.9%) | 0 (0%) | 0 (0%) | 0 (0%) | 0 (0%) |
|  | 0 (0%) | 0 (0%) | 0 (0%) | 0 (0%) | 0 (0%) | 0 (0%) | 0 (0%) | 0 (0%) | 1 (5.3%) | 0 (0%) |
|  | 0 (0%) | 0 (0%) | 0 (0%) | 0 (0%) | 0 (0%) | 1 (2.9%) | 0 (0%) | 0 (0%) | 0 (0%) | 0 (0%) |
|  | 0 (0%) | 0 (0%) | 0 (0%) | 0 (0%) | 1 (3.8%) | 0 (0%) | 0 (0%) | 0 (0%) | 0 (0%) | 0 (0%) |
|  | 0 (0%) | 0 (0%) | 0 (0%) | 0 (0%) | 1 (3.8%) | 0 (0%) | 0 (0%) | 0 (0%) | 0 (0%) | 0 (0%) |
|  | 0 (0%) | 0 (0%) | 0 (0%) | 0 (0%) | 0 (0%) | 1 (2.9%) | 0 (0%) | 0 (0%) | 0 (0%) | 0 (0%) |
|  | 0 (0%) | 0 (0%) | 0 (0%) | 0 (0%) | 1 (3.8%) | 0 (0%) | 0 (0%) | 0 (0%) | 0 (0%) | 0 (0%) |
|  | 0 (0%) | 0 (0%) | 0 (0%) | 0 (0%) | 0 (0%) | 1 (2.9%) | 0 (0%) | 0 (0%) | 0 (0%) | 0 (0%) |
|  | 0 (0%) | 0 (0%) | 0 (0%) | 0 (0%) | 1 (3.8%) | 0 (0%) | 0 (0%) | 0 (0%) | 0 (0%) | 0 (0%) |
|  | 0 (0%) | 0 (0%) | 0 (0%) | 0 (0%) | 0 (0%) | 1 (2.9%) | 0 (0%) | 0 (0%) | 0 (0%) | 0 (0%) |
|  | 0 (0%) | 0 (0%) | 0 (0%) | 0 (0%) | 1 (3.8%) | 0 (0%) | 0 (0%) | 0 (0%) | 0 (0%) | 0 (0%) |
|  | 0 (0%) | 0 (0%) | 0 (0%) | 0 (0%) | 1 (3.8%) | 0 (0%) | 0 (0%) | 0 (0%) | 0 (0%) | 0 (0%) |
|  | 0 (0%) | 0 (0%) | 0 (0%) | 0 (0%) | 1 (3.8%) | 0 (0%) | 0 (0%) | 0 (0%) | 0 (0%) | 0 (0%) |
|  | 0 (0%) | 0 (0%) | 0 (0%) | 0 (0%) | 0 (0%) | 0 (0%) | 0 (0%) | 0 (0%) | 0 (0%) | 1 (10%) |
|  | 0 (0%) | 0 (0%) | 0 (0%) | 0 (0%) | 0 (0%) | 1 (2.9%) | 0 (0%) | 0 (0%) | 0 (0%) | 0 (0%) |
|  | 0 (0%) | 0 (0%) | 0 (0%) | 0 (0%) | 0 (0%) | 1 (2.9%) | 0 (0%) | 0 (0%) | 0 (0%) | 0 (0%) |
|  | 0 (0%) | 0 (0%) | 0 (0%) | 0 (0%) | 1 (3.8%) | 0 (0%) | 0 (0%) | 0 (0%) | 0 (0%) | 0 (0%) |
|  | 0 (0%) | 0 (0%) | 0 (0%) | 0 (0%) | 1 (3.8%) | 0 (0%) | 0 (0%) | 0 (0%) | 0 (0%) | 0 (0%) |
|  | 0 (0%) | 0 (0%) | 0 (0%) | 0 (0%) | 0 (0%) | 0 (0%) | 0 (0%) | 0 (0%) | 0 (0%) | 1 (10%) |
|  | 0 (0%) | 0 (0%) | 0 (0%) | 0 (0%) | 0 (0%) | 0 (0%) | 0 (0%) | 0 (0%) | 1 (5.3%) | 0 (0%) |
|  | 0 (0%) | 0 (0%) | 0 (0%) | 0 (0%) | 0 (0%) | 1 (2.9%) | 0 (0%) | 0 (0%) | 0 (0%) | 0 (0%) |
|  | 1 (9.1%) | 0 (0%) | 0 (0%) | 0 (0%) | 0 (0%) | 0 (0%) | 0 (0%) | 0 (0%) | 0 (0%) | 0 (0%) |
|  | 0 (0%) | 0 (0%) | 0 (0%) | 0 (0%) | 0 (0%) | 0 (0%) | 0 (0%) | 0 (0%) | 1 (5.3%) | 0 (0%) |
| ihc16 |  |  |  |  |  |  |  |  |  |  |
| Mild | 5 (45%) | 0 (0%) | 0 (0%) | 0 (0%) | 18 (69%) | 5 (15%) | 0 (0%) | 0 (0%) | 2 (11%) | 4 (40%) |
| Moderate | 0 (0%) | 1 (50%) | 0 (0%) | 0 (0%) | 3 (12%) | 20 (59%) | 1 (100%) | 0 (0%) | 1 (5.3%) | 2 (20%) |
| Negative | 6 (55%) | 1 (50%) | 1 (100%) | 3 (75%) | 5 (19%) | 4 (12%) | 0 (0%) | 1 (100%) | 7 (37%) | 4 (40%) |
| Strong | 0 (0%) | 0 (0%) | 0 (0%) | 1 (25%) | 0 (0%) | 5 (15%) | 0 (0%) | 0 (0%) | 9 (47%) | 0 (0%) |
| ihc18 |  |  |  |  |  |  |  |  |  |  |
| Mild | 3 (27%) | 0 (0%) | 0 (0%) | 0 (0%) | 6 (23%) | 3 (8.8%) | 1 (100%) | 1 (100%) | 5 (26%) | 5 (50%) |
| Moderate | 1 (9.1%) | 1 (50%) | 0 (0%) | 0 (0%) | 0 (0%) | 4 (12%) | 0 (0%) | 0 (0%) | 2 (11%) | 1 (10%) |
| Negative | 7 (64%) | 1 (50%) | 1 (100%) | 4 (100%) | 20 (77%) | 24 (71%) | 0 (0%) | 0 (0%) | 10 (53%) | 2 (20%) |
| Strong | 0 (0%) | 0 (0%) | 0 (0%) | 0 (0%) | 0 (0%) | 3 (8.8%) | 0 (0%) | 0 (0%) | 2 (11%) | 2 (20%) |
| pcr16 |  |  |  |  |  |  |  |  |  |  |
| Negative | 7 (64%) | 1 (50%) | 0 (0%) | 2 (50%) | 3 (12%) | 6 (18%) | 0 (0%) | 1 (100%) | 9 (47%) | 2 (20%) |
| Positive | 4 (36%) | 1 (50%) | 1 (100%) | 2 (50%) | 23 (88%) | 28 (82%) | 1 (100%) | 0 (0%) | 10 (53%) | 8 (80%) |
| pcr18 |  |  |  |  |  |  |  |  |  |  |
| Negative | 3 (75%) | 1 (50%) | 1 (100%) | 4 (100%) | 2 (40%) | 5 (45%) | 1 (100%) | 0 (NA%) | 11 (65%) | 1 (25%) |
| Positive | 1 (25%) | 1 (50%) | 0 (0%) | 0 (0%) | 3 (60%) | 6 (55%) | 0 (0%) | 0 (NA%) | 6 (35%) | 3 (75%) |
| Unknown | 7 | 0 | 0 | 0 | 21 | 23 | 0 | 1 | 2 | 6 |
| x8 |  |  |  |  |  |  |  |  |  |  |
| Negative | 3 (43%) | 0 (NA%) | 0 (NA%) | 0 (NA%) | 18 (86%) | 20 (87%) | 0 (NA%) | 0 (0%) | 2 (100%) | 0 (0%) |
| Positive | 4 (57%) | 0 (NA%) | 0 (NA%) | 0 (NA%) | 3 (14%) | 3 (13%) | 0 (NA%) | 1 (100%) | 0 (0%) | 6 (100%) |
| Unknown | 4 | 2 | 1 | 4 | 5 | 11 | 1 | 0 | 17 | 4 |
| diagnosis_score | 1.00 ± 0.00 | 2.00 ± 0.00 | 3.00 ± NA | 4.00 ± 0.00 | 5.00 ± 0.00 | 6.00 ± 0.00 | 7.00 ± NA | 8.00 ± NA | 9.00 ± 0.00 | 10.00 ± 0.00 |
| ihc16_num |  |  |  |  |  |  |  |  |  |  |
| 0 | 6 (55%) | 1 (50%) | 1 (100%) | 3 (75%) | 5 (19%) | 4 (12%) | 0 (0%) | 1 (100%) | 7 (37%) | 4 (40%) |
| 1 | 5 (45%) | 0 (0%) | 0 (0%) | 0 (0%) | 18 (69%) | 5 (15%) | 0 (0%) | 0 (0%) | 2 (11%) | 4 (40%) |
| 2 | 0 (0%) | 1 (50%) | 0 (0%) | 0 (0%) | 3 (12%) | 20 (59%) | 1 (100%) | 0 (0%) | 1 (5.3%) | 2 (20%) |
| 3 | 0 (0%) | 0 (0%) | 0 (0%) | 1 (25%) | 0 (0%) | 5 (15%) | 0 (0%) | 0 (0%) | 9 (47%) | 0 (0%) |
| ihc18_num |  |  |  |  |  |  |  |  |  |  |
| 0 | 7 (64%) | 1 (50%) | 1 (100%) | 4 (100%) | 20 (77%) | 24 (71%) | 0 (0%) | 0 (0%) | 10 (53%) | 2 (20%) |
| 1 | 3 (27%) | 0 (0%) | 0 (0%) | 0 (0%) | 6 (23%) | 3 (8.8%) | 1 (100%) | 1 (100%) | 5 (26%) | 5 (50%) |
| 2 | 1 (9.1%) | 1 (50%) | 0 (0%) | 0 (0%) | 0 (0%) | 4 (12%) | 0 (0%) | 0 (0%) | 2 (11%) | 1 (10%) |
| 3 | 0 (0%) | 0 (0%) | 0 (0%) | 0 (0%) | 0 (0%) | 3 (8.8%) | 0 (0%) | 0 (0%) | 2 (11%) | 2 (20%) |
| pcr16_num | 4 (36%) | 1 (50%) | 1 (100%) | 2 (50%) | 23 (88%) | 28 (82%) | 1 (100%) | 0 (0%) | 10 (53%) | 8 (80%) |
| pcr18_num | 1 (25%) | 1 (50%) | 0 (0%) | 0 (0%) | 3 (60%) | 6 (55%) | 0 (0%) | 0 (NA%) | 6 (35%) | 3 (75%) |
| Unknown | 7 | 0 | 0 | 0 | 21 | 23 | 0 | 1 | 2 | 6 |
| ihc16_binary |  |  |  |  |  |  |  |  |  |  |
| Negative | 6 (55%) | 1 (50%) | 1 (100%) | 3 (75%) | 5 (19%) | 4 (12%) | 0 (0%) | 1 (100%) | 7 (37%) | 4 (40%) |
| Positive | 5 (45%) | 1 (50%) | 0 (0%) | 1 (25%) | 21 (81%) | 30 (88%) | 1 (100%) | 0 (0%) | 12 (63%) | 6 (60%) |
| ihc18_binary |  |  |  |  |  |  |  |  |  |  |
| Negative | 7 (64%) | 1 (50%) | 1 (100%) | 4 (100%) | 20 (77%) | 24 (71%) | 0 (0%) | 0 (0%) | 10 (53%) | 2 (20%) |
| Positive | 4 (36%) | 1 (50%) | 0 (0%) | 0 (0%) | 6 (23%) | 10 (29%) | 1 (100%) | 1 (100%) | 9 (47%) | 8 (80%) |
| pcr16_binary |  |  |  |  |  |  |  |  |  |  |
| Negative | 7 (64%) | 1 (50%) | 0 (0%) | 2 (50%) | 3 (12%) | 6 (18%) | 0 (0%) | 1 (100%) | 9 (47%) | 2 (20%) |
| Positive | 4 (36%) | 1 (50%) | 1 (100%) | 2 (50%) | 23 (88%) | 28 (82%) | 1 (100%) | 0 (0%) | 10 (53%) | 8 (80%) |
| pcr18_binary |  |  |  |  |  |  |  |  |  |  |
| Negative | 3 (75%) | 1 (50%) | 1 (100%) | 4 (100%) | 2 (40%) | 5 (45%) | 1 (100%) | 0 (NA%) | 11 (65%) | 1 (25%) |
| Positive | 1 (25%) | 1 (50%) | 0 (0%) | 0 (0%) | 3 (60%) | 6 (55%) | 0 (0%) | 0 (NA%) | 6 (35%) | 3 (75%) |
| Unknown | 7 | 0 | 0 | 0 | 21 | 23 | 0 | 1 | 2 | 6 |
| ^1^Mean ± SD; n (%) | | | | | | | | | | |

| Age | DIAGNOSIS | IHC HPV 16-E7 | IHC HPV 18-E7 | PCR HPV 16 | PCR HPV 18 | |
| --- | --- | --- | --- | --- | --- | --- |
| 58 | AC | Moderate | Strong | Positive | Positive | |
| 50 | HSIL | Negative | Negative | Negative | Positive | |
| 34 | Squamous metaplasia | Moderate | Moderate | Positive | Negative | |
| 39 | LSIL | Mild | Negative | Positive | Negative | |
| 67 | SCC | Strong | Negative | Positive | Negative | |
| 63 | SCC | Negative | Strong | Negative | Positive | |
| 51 | ASC | Negative | Negative | Positive | Negative | |
| 56 | HSIL | Strong | Strong | Positive | Positive | |
| 43 | ACIS | Moderate | Mild | Positive | Negative | |
| 62 | SCC | Negative | Negative | Negative | Negative | |
| 68 | SCC | Negative | Negative | Negative | Negative | |
| 57 | HSIL | Strong | Negative | Positive | Negative | |
| 63 | SCC | Negative | Mild | Negative | Positive | |
| 61 | AC | Negative | Mild | Negative | Negative | |
| 62 | SCC | Mild | Moderate | Negative | Positive | |
| 59 | HSIL | Negative | Strong | Negative | Positive | |
| 54 | HSIL | Mild | Mild | Positive | Positive | |
| 55 | AC | Mild | Strong | Positive | Positive | |
| 64 | SCC | Strong | Mild | Positive | Negative | |
| 62 | SCC | Negative | Mild | Negative | Positive | |
| 64 | SCC | Negative | Negative | Negative | Negative | |
| 60 | AC | Negative | Mild | Negative | Positive | |
| 55 | HSIL | Moderate | Moderate | Positive | Positive | |
| 35 | LSIL | Moderate | Mild | Positive | Positive | |
| 41 | HSIL | Strong | Strong | Positive | Positive | |
| 48 | LSIL | Mild | Mild | Positive | Positive | |
| 40 | ASC | Strong | Negative | Positive | Negative | |
| 44 | LSIL | Mild | Negative | Positive | Negative | |
| 38 | ASC | Negative | Negative | Negative | Negative | |
| 62 | SCC | Strong | Negative | Positive | Negative | |
| 54 | HSIL | Strong | Mild | Positive | Negative | |
| 59 | HSIL | Moderate | Negative | Positive | Negative | |
| 38 | NILM | Mild | Negative | Negative | Negative | |
| 56 | NILM | Negative | Negative | Negative | Negative | |
| 65 | SCC | Strong | Mild | Positive | Negative | |
| 61 | SCC | Strong | Negative | Positive | Negative | |
| 57 | HSIL | Moderate | Negative | Positive | Negative | |
| 40 | ASC | Negative | Negative | Negative | Negative | |
| 63 | SCC | Mild | Moderate | Negative | Positive | |
| 64 | SCC | Moderate | Negative | Positive | Negative | |
| 42 | LSIL | Mild | Mild | Positive | Positive | |
| 60 | SCC | Strong | Negative | Positive | Negative | |
| 65 | SCC | Negative | Strong | Negative | Positive | |
| 33 | Koilocytic change | Negative | Negative | Positive | Negative | |
| 45 | NILM | Negative | Negative | Negative | Negative | |
| 31 | Squamous metaplasia | Negative | Negative | Negative | Positive | |
| 38 | NILM | Mild | Moderate | Positive | Positive | |
| 47 | HSIL | Negative | Negative | Negative | Negative | |
| 59 | SCC | Strong | Negative | Positive | Negative | |
| 30 | NILM | Mild | Negative | Positive | | Negative |
| 37 | NILM | Negative | Mild | Negative | | Positive |
| 56 | HSIL | Negative | Moderate | Negative | | Positive |
| 42 | LSIL | Mild | Negative | Positive | | Negative |
| 53 | HSIL | Moderate | Negative | Positive | | Negative |
| 59 | HSIL | Moderate | Negative | Positive | | Negative |
| 51 | HSIL | Mild | Moderate | Negative | | Positive |
| 53 | HSIL | Moderate | Negative | Positive | | Negative |
| 50 | AC | Mild | Mild | Positive | | Positive |
| 62 | HSIL | Moderate | Mild | Positive | | Negative |
| 60 | HSIL | Moderate | Negative | Positive | | Negative |
| 55 | HSIL | Strong | Negative | Positive | | Negative |
| 46 | NILM | Negative | Negative | Negative | | Positive |
| 66 | AC | Negative | Negative | Positive | | Positive |
| 40 | LSIL | Mild | Negative | Positive | | Negative |
| 38 | NILM | Mild | Negative | Positive | | Negative |
| 52 | HSIL | Moderate | Negative | Positive | | Negative |
| 50 | LSIL | Mild | Negative | Positive | | Negative |
| 43 | LSIL | Mild | Negative | Positive | | Negative |
| 56 | HSIL | Moderate | Negative | Positive | | Negative |
| 58 | HSIL | Mild | Negative | Positive | | Negative |
| 60 | HSIL | Moderate | Negative | Positive | | Negative |
| 53 | LSIL | Mild | Negative | Positive | | Negative |
| 53 | HSIL | Moderate | Negative | Positive | | Negative |
| 54 | LSIL | Mild | Negative | Positive | | Negative |
| 38 | NILM | Negative | Mild | Negative | | Positive |
| 32 | LSIL | Negative | Mild | Negative | | Positive |
| 36 | LSIL | Negative | Negative | Positive | | Negative |
| 53 | HSIL | Moderate | Negative | Positive | | Negative |
| 40 | HSIL | Mild | Moderate | Negative | | Positive |
| 58 | AC | Moderate | Moderate | Positive | | Positive |
| 40 | LSIL | Mild | Negative | Positive | | Negative |
| 37 | LSIL | Mild | Negative | Positive | | Negative |
| 45 | LSIL | Mild | Negative | Positive | | Negative |
| 39 | NILM | Negative | Mild | Negative | | Positive |
| 40 | LSIL | Negative | Negative | Positive | | Negative |
| 62 | AC | Mild | Mild | Positive | | Positive |
| 47 | AIS | Negative | Mild | Negative | | Positive |
| 50 | HSIL | Moderate | Negative | Positive | | Negative |
| 54 | HSIL | Moderate | Negative | Positive | | Negative |
| 43 | LSIL | Moderate | Negative | Positive | | Negative |
| 48 | LSIL | Mild | Negative | Positive | | Negative |
| 54 | HSIL | Moderate | Negative | Positive | | Negative |
| 39 | LSIL | Moderate | Negative | Positive | | Negative |
| 50 | HSIL | Mild | Negative | Positive | | Negative |
| 44 | LSIL | Negative | Mild | Negative | | Positive |
| 49 | HSIL | Moderate | Negative | Positive | | Negative |
| 36 | LSIL | Mild | Negative | Positive | | Negative |
| 41 | LSIL | Mild | Negative | Positive | | Negative |
| 44 | LSIL | Mild | Negative | Positive | | Negative |
| 60 | AC | Negative | Negative | Positive | | Positive |
| 64 | HSIL | Moderate | Negative | Positive | | Negative |
| 47 | HSIL | Moderate | Negative | Positive | | Negative |
| 33 | LSIL | Negative | Mild | Negative | | Positive |
| 47 | LSIL | Mild | Negative | Positive | | Negative |
| 68 | AC | Mild | Mild | Positive | | Positive |
| 61 | SCC | Strong | Mild | Positive | | Negative |
| 54 | HSIL | Moderate | Negative | Positive | | Negative |
| 41 | NILM | Mild | Negative | Positive | | Negative |
| 59 | SCC | Strong | Negative | Positive | | Negative |
